# Development and Bayesian Uncertainty Quantification of Transferable Coarse-Grained Models for Carbohydrates

**DOI:** 10.1021/acs.jctc.6c01202

**Published:** 2026-10-08

**Authors:** Parisa Farzeen, Soumil Y. Joshi, Abhishek T. Sose, Sanket A. Deshmukh

**Affiliations:** Department of Chemical Engineering, 1757 Virginia Tech, Blacksburg, Virginia 24061, United States

## Abstract

Carbohydrates play an important role in industrial, biomedical, and materials applications. The exceptional structural diversity of carbohydrates makes their accurate molecular modeling both essential and challenging. Although coarse-grained (CG) molecular dynamics (MD) simulations provide access to large length and time scales relevant to carbohydrate assembly and materials behavior, the development of uncertainty-quantified transferable CG models remains challenging and limited. In this work, we employ a computational framework that integrates multiscale modeling, optimization algorithms, and machine learning to develop CG models that reproduce the structural, physical, and thermodynamic properties of 12 pyranose monosaccharides. To preserve their stereochemical and geometric features while balancing computational efficiency, each monosaccharide is represented using a six-bead mapping scheme, with each CG bead mapped based on the monosaccharide functional groups. To ensure transferability, force field (FF) parameters for CG models of three model monosaccharides, α-d-glucose, β-d-xylose, and α-l-rhamnose, were optimized using Particle Swarm Optimization (PSO). Bayesian uncertainty quantification was subsequently performed to assess the robustness of the optimized models by quantifying uncertainty in both the model parameters and the predicted properties. To assess parameter transferability, we applied the optimized CG beads developed for the model monosaccharides to other monosaccharides in CG MD simulations. The resulting CG models accurately reproduced thermodynamic and structural properties across 12 monosaccharides, demonstrating good transferability. Finally, the predictive capability of the optimized CG parameters was further assessed across 12 monosaccharides using glass transition temperatures and surface energies, which were not included during parameter optimization. The model reproduced glass transition temperature with good accuracy and captured qualitative surface-energy trends, demonstrating predictive capability beyond the optimized properties. Overall, this work establishes an uncertainty-aware CG modeling framework that enhances confidence in CG simulations and provides a scalable foundation for extending to oligosaccharides, polysaccharides, and hydrated carbohydrate assemblies.

## Graphical abstract

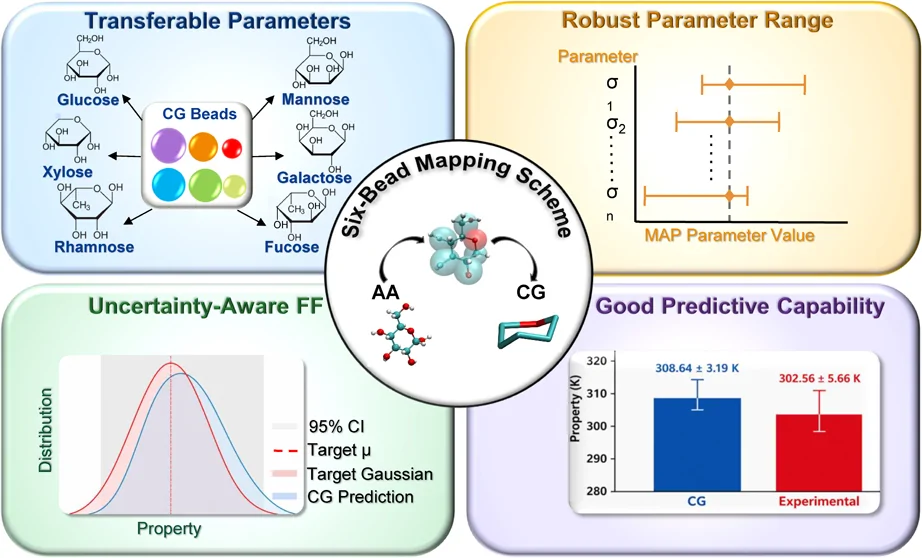

## 1 Introduction

Carbohydrates are among nature’s most abundant and versatile biomolecules, playing key roles in energy storage, structural support, molecular recognition, and a host of related biological processes.^1–3^ This functional diversity stems from their remarkable chemical tunability, small variations in glycosidic linkage, stereochemistry, and branching drive pronounced changes in solvation, hydration, intermolecular interactions, and self-assembly.^4–11^ Coupled with their broadly favorable biocompatibility and biodegradability, these features establish carbohydrates as prime candidates for biomedical and materials applications.^12–16^ Resolving how molecular architecture dictates material-level behavior is therefore central to the rational design of carbohydrate-based systems.

Despite extensive experimental efforts, establishing clear links between these molecular features and macroscopic properties such as solubility, viscosity, and phase behavior remains difficult, owing to the intrinsic flexibility and structural heterogeneity of carbohydrates.^17–21^ This challenge has motivated the use of molecular simulations to complement experiments with molecular-level insight. Molecular dynamics (MD) simulations, built on force field (FF) parameters that describe bonded and nonbonded interactions, have proven particularly valuable for probing glycosidic linkage preferences, anomeric and exoanomeric effects, and hydration-driven conformational behavior.^22–27^ Although all-atom (AA) MD provides detailed chemical resolution, its computational cost limits access to the length and time scales relevant to mesoscale structure formation and self-assembly. This limitation has driven the development of coarse-grained (CG) models, in which groups of atoms are represented as interaction sites or beads. By reducing the number of degrees of freedom and smoothing the underlying energy landscape, CG models enable simulations of larger systems over longer time scales.^28,29^

Despite the structural diversity of carbohydrates and the limited availability of systematic experimental benchmarks, considerable effort has been devoted to developing CG models that reproduce available structural and thermodynamic properties.^30–33^ The M3B model for α(1 → 4) d-glucans, which assigns three CG beads per glucose, successfully captures the helical conformations of oligosaccharides as well as experimentally reported glass transition temperatures.^30^ Building on this three-bead mapping, Liu et al. derived a multiscale coarse-grained (MS-CG) model of α-d-glucopyranose in water directly from AA simulations, reproducing structural and thermodynamic properties of the underlying atomistic system.^32^ Complementary system-specific approaches have also been developed for more complex carbohydrates. For example, Bathe et al. took a complementary approach for glycosaminoglycans (GAGs), chondroitin (CH), chondroitin sulfate (CS), and hyaluronic acid (HA), using an even coarser per-residue mapping in which AA simulations of disaccharides generated pre-tabulated glycosidic torsion potentials, with electrostatic and steric interactions incorporated through virtual sites.^33^ The resulting model reproduced experimental radii of gyration and end-to-end distances with quantitative accuracy.^33^ More general frameworks have emerged through Martini-based carbohydrate models. López et al. extended the Martini FF to carbohydrates by representing each monosaccharide with three CG beads, with parameters guided by conformational sampling of small carbohydrates against AA references and further refined using octanol-water partitioning free energies to assign appropriate bead types.^20^ Their model reproduced water-octanol partitioning in agreement with both AA simulations and experiment while capturing the conformational and dynamic behavior of CG amylose and curdlan consistent with atomistic results.^20^ Grünewald et al. introduced a systematic strategy within Martini 3 to parametrize arbitrary carbohydrates via a canonical fragment-based mapping at the same three-bead-per-sugar resolution, reproducing the osmotic pressures of carbohydrate-water solutions and correctly distinguishing the solubility behavior of dextran from that of cellulose.^34^ Building on the Martini carbohydrate FF,^20^ Wohlert et al. retained this three-bead mapping but tailored the nonbonded interactions to model crystalline cellulose, obtaining a stable ordered structure closely resembling cellulose Iβ in aqueous environments.^35^ Collectively, these studies have substantially advanced carbohydrate CG modeling across both system-specific and general frameworks. However, many existing models rely on relatively coarse mappings, often near three beads per monosaccharide, which can limit the representation of fine stereochemical, linkage-specific, and conformational features. Consequently, no single CG framework has yet simultaneously achieved broad transferability across diverse carbohydrate chemistries, robust performance across target properties, and accurate preservation of key molecular-level structural signatures.

A critical and largely unaddressed limitation of existing CG carbohydrate FFs is the lack of rigorous quantification of parameter and property uncertainty. FF parameters are typically reported as single optimized values, despite uncertainties arising from limited training data, model approximations, and parameter correlations. Because MD predictions can depend sensitively on these parameters, the absence of uncertainty estimates limits confidence in model robustness and transferability, which remain central challenges for carbohydrate CG modeling. Recent work by our group demonstrated that Bayesian uncertainty quantification (BUQ) provides a principled framework for characterizing uncertainty in FF parameters, with posterior distributions capturing parameter correlations, confidence intervals, and the range of physically plausible parameter sets consistent with available data.^36^ In the present work, Particle Swarm Optimization (PSO) and BUQ therefore serve sequential and complementary purposes. PSO is used as the objective-function-based optimizer to identify a nominal best-fit set of CG parameters that reproduces the selected AA-derived property targets. BUQ is then coupled with machine-learning (ML) surrogate models and subsequently applied to a local parameter space centered on the PSO solution. Rather than re-optimizing the FF, BUQ efficiently samples parameter space, allowing CG FF parameters to be inferred as statistically meaningful distributions rather than single optimized values. This approach also enables systematic assessment of parameter sensitivity and predictive reliability.

Here, we develop transferable CG models for pyranose monosaccharides that address the three challenges identified above: transferability across diverse carbohydrate chemistries, accurate reproduction of key structural and thermodynamic properties, and robustness through Bayesian quantification of parameter uncertainty. The pyranose ring is the dominant ring form for many common carbohydrates in aqueous solution and is generally energetically favored,^37,38^ making it a physically relevant starting point for CG model development. Because monosaccharides are the fundamental structural units of oligo- and polysaccharides, we first develop and validate CG representations at the monosaccharide level against mapped AA reference trajectories. This hierarchical strategy provides a controlled foundation for future extension to larger carbohydrate systems.

First, we use a hybrid bottom-up/top-down parameterization strategy in which bonded interactions are derived from structural distributions obtained from AA reference trajectories of solvent-free, condensed-phase bulk monosaccharide systems, while nonbonded interactions are optimized to reproduce the bulk density and heat of vaporization (H_v_) of the same systems.^39–42^ This allows the models to capture both microscopic conformational behavior and thermodynamic properties. This choice of reference state, which is distinct from the solvated and aqueous-thermodynamic targets used in prior CG carbohydrate FFs, is detailed and justified in Section 2.1. Second, each monosaccharide is represented using a six-bead mapping that preserves molecular geometry and key stereochemical features while remaining computationally efficient for larger-scale simulations. Third, chemically transferable bead types represent axial and equatorial carbon environments, with additional bead types introduced as needed to distinguish among monosaccharide identities. This design enables the framework to discriminate among different monosaccharides while preserving transferability. Fourth, all CG beads are charge-neutral, which accelerates simulations by avoiding explicit electrostatic calculations while retaining key structural and thermodynamic behavior. Fifth, the parameter-optimization and uncertainty-analysis stages are explicitly separated. PSO provides the nominal best-fit nonbonded parameter set, whereas BUQ characterizes confidence in that solution and identifies the surrounding parameter combinations that remain statistically consistent with the target properties.

Collectively, these features yield a transferable CG framework that captures essential stereochemical, structural, and thermodynamic behavior of pyranose monosaccharides while remaining computationally efficient for future simulations of larger carbohydrate systems.

## 2 Methodology

The development of CG models for pyranose monosaccharides proceeded through three stages: (i) CG FF Parameter Optimization, (ii) Uncertainty Quantification and Robustness Assessment, and (iii) Transferability Evaluation. The overall computational workflow is illustrated in Figure 1.

**Figure 1:**
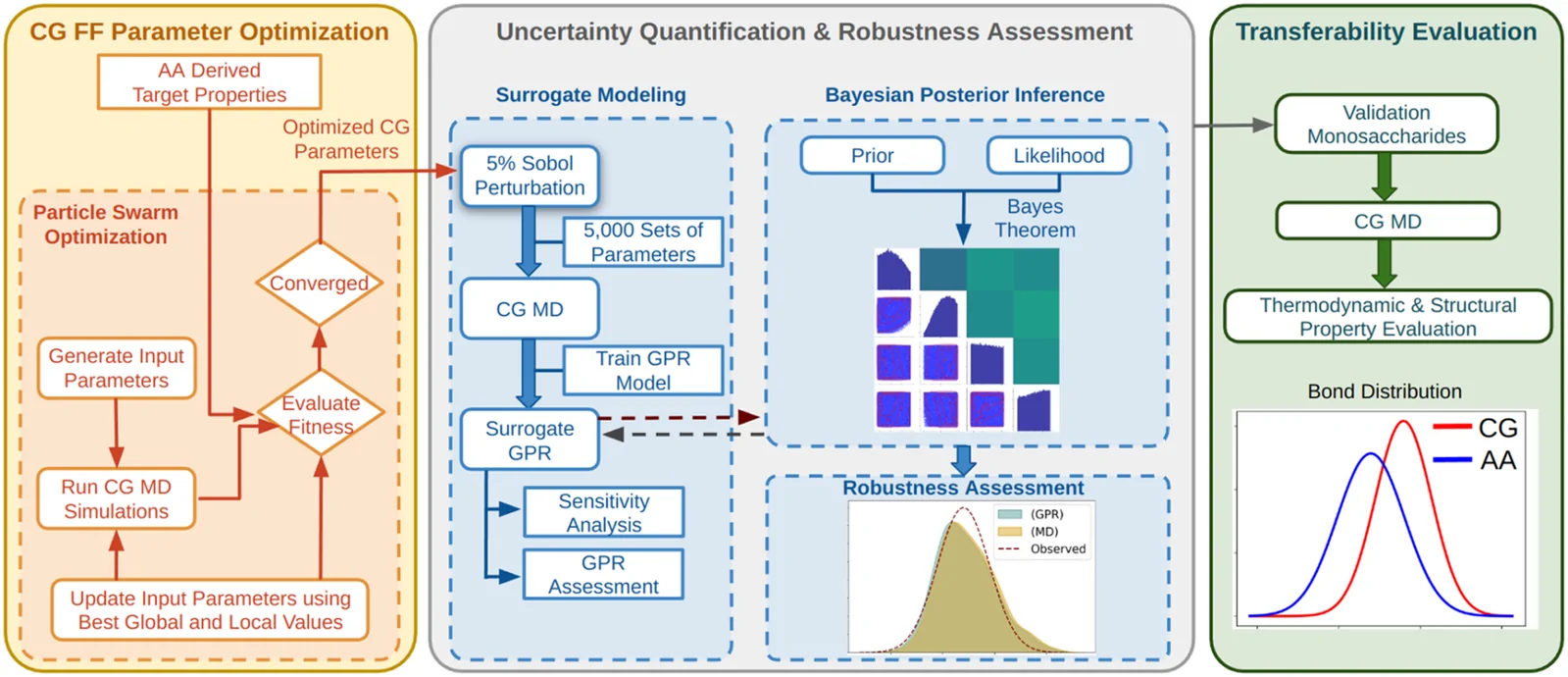
Framework showing the CG model development and uncertainty quantification of carbohydrates. **CG Model Development:** Particle Swarm Optimization (PSO) was coupled with CG MD simulations to obtain optimal CG parameters. **Uncertainty Quantification and Robustness Assessment:** These optimal parameters were perturbed by 5% using Sobol sampling to generate 5000 new parameter sets for further CG MD simulations. Resulting sets were used to train a Gaussian Process Regression (GPR) model, which acted as a surrogate for CG MD in BUQ analysis to compute robust posteriors. **Transferability Evaluation:** Optimized parameters were tested on 9 different validation monosaccharides to evaluate the transferability of the CG model.

### 2.1 CG FF Parameter Optimization

Prior CG carbohydrate FFs derive their parameters from reference systems in which water is explicitly present, either through AA reference trajectories of solvated sugars as in the MS-CG framework of Liu et al.^32^ or through thermodynamic targets measured in aqueous and water-octanol environments as in the Martini carbohydrate models of López et al.^20^ and Grünewald et al.^34^ Water therefore mediates the local environment of every reference sugar, and the effective sugar–sugar interaction inferred during parameter optimization reflects water-mediated correlations rather than direct sugar–sugar contacts.

We instead optimize CG parameters against solvent-free, condensed-phase bulk AA reference simulations of the pure monosaccharide.^43,44^ The nonbonded parameters are tuned to reproduce the reference AA bulk density and heat of vaporization (H_v_), which are by definition properties of the condensed phase and therefore require a bulk reference state.^45^ The purpose of excluding water at this stage is to isolate and accurately capture the effective sugar-sugar interactions without the additional influence of solvent-mediated effects.

#### 2.1.1 Mapping Scheme

To develop transferable CG models for pyranose monosaccharides, we introduced a mapping scheme that preserves the ring geometry across different monosaccharide types, as shown in Figures 2 and S1. Three monosaccharides, α-d-glucose, β-d-xylose, and α-l-rhamnose, were selected for parameter optimization and are referred to as the model monosaccharides throughout the remainder of this article. These molecules were chosen to enable transferable parameterization of all proposed CG bead types while spanning representative hexopyranose, pentopyranose, and deoxyhexopyranose chemistries.

**Figure 2:**
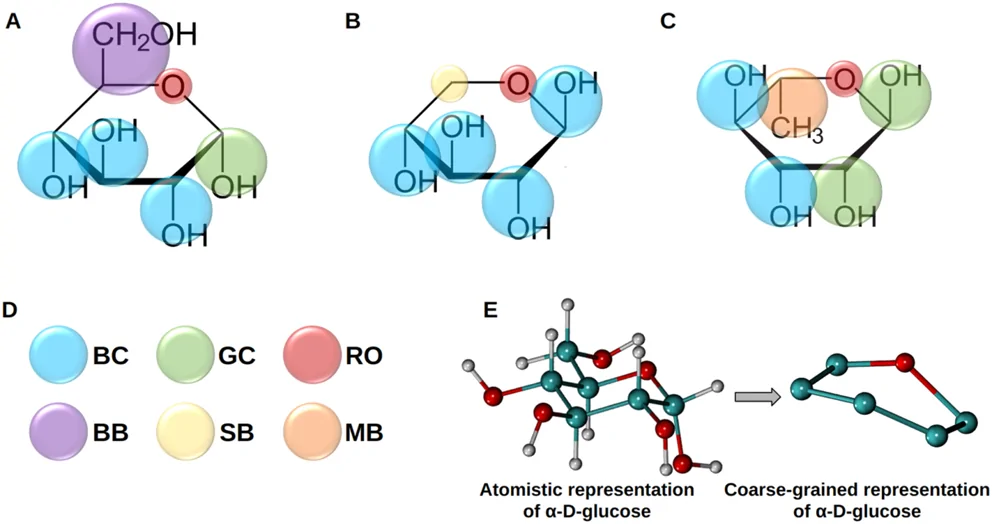
AA structures of monosaccharides with mapped CG beads, (A) α-d-glucose, (B) β-d-xylose, and (C) α-l-rhamnose. (D) shows the type of CG beads used, and (E) shows AA and CG representations of α-d-glucose.

All monosaccharides share three common bead types. The GC and BC beads use a 2:1 mapping and distinguish between axial and equatorial stereochemical environments within the pyranose ring, respectively, while the RO bead represents the ring oxygen atom using a 1:1 mapping (Figure 2). Specifically, these bead types represent whether carbon-centered −OH groups are oriented approximately perpendicular (axial, shown with GC beads) or approximately within the plane (equatorial, shown with BC beads) of the ring, thereby preserving key geometric features of carbohydrate stereochemistry. Additional bead types were introduced to distinguish monosaccharide-specific structural features. The BB bead, with a 3:1 mapping, was developed for hexopyranose monosaccharides with molecular formula C_6_H_12_O_6_, while the MB bead, with a 2:1 mapping, was introduced for deoxyhexopyranose monosaccharides with molecular formula C_6_H_12_O_5_. For the pentopyranose xylose, the SB bead provides a 1:1 mapping. These bead types were extended to nine additional monosaccharides, referred to as validation monosaccharides, as shown in Figure S1. These validation monosaccharides were used to evaluate model transferability, as discussed in Section 2.3.

The atomistic constituents and mass of each bead are listed in Table S1. Beads are further designated as D or L (DGC/LGC, DBC, LBC, DRO/LRO, DBB, DSB, LMB), as summarized in Table S1, to distinguish stereochemical environments associated with d- and l-sugar stereoisomers. Details of the procedure used to obtain CG bead positions and equilibrium bond lengths, angles, and dihedrals are provided in Section S1.1 of the Supporting Information (SI).

All CG beads were assigned zero partial charge, thereby avoiding explicit long-range electrostatic calculations in the CG simulations. To account for the net effects of electrostatic interactions at the mapped resolution, the Lennard-Jones (LJ) parameters were optimized against thermodynamic targets that implicitly reflect these interactions. The CHARMM-type FF functional form used to describe bonded and nonbonded interactions between CG beads is given in eq 1,^46^

$$U=\sum k_{b}{\left(b-b_{o}\right)}^{2}+\sum k_{\theta }{\left(\theta -\theta_{0}\right)}^{2}+\sum k_{\Phi }\left(1+\cos\left(n\Phi -\delta \right)\right)+4\epsilon_{\mathrm{ij}}\left[{\left(\frac{\sigma_{\mathrm{ij}}}{r_{\mathrm{ij}}}\right)}^{12}-{\left(\frac{\sigma_{\mathrm{ij}}}{r_{\mathrm{ij}}}\right)}^{6}\right]$$ 1

The first three terms describe bonded interactions, harmonic bond and angle potentials, with force constants *k*_b_ and *k*_θ_, and equilibrium values *b*_0_ and θ_0_, and a cosine dihedral potential. In the dihedral term, Φ is the angle between the planes defined by adjacent triplets of four consecutively bonded beads, n is the multiplicity, and δ is the phase. Nonbonded interactions are described using a 12-6 LJ potential, where ϵ is the depth of the potential well, σ is the finite distance at which the inter-bead potential is zero, and *r_ij_* is the distance between beads *i* and *j*.^47^ Note, in eq 1, ϵ denotes the positive magnitude of the LJ well depth. However, CHARMM-format parameter files report the corresponding potential minimum as *E*_min_ = −ϵ. Accordingly, the nonbonded energy parameters listed in the tables are reported using the CHARMM sign convention. Cross-interactions between different bead types were calculated using the Lorentz-Berthelot (LB) mixing rule.^48^ Together, this mapping and functional form preserve the geometrical structure of monosaccharides while enabling discrimination among sugar types through bead-specific bonded and nonbonded parameters.

#### 2.1.2 Simulation Details

All AA and CG MD simulations were performed using the Nanoscale Molecular Dynamics (NAMD) 2.14 program.^49^ Initial three-dimensional AA structures of individual monosaccharides were obtained in Protein Data Bank (PDB) format from the CHARMM Small Molecule Library (CSML).^50^ Bulk systems were then generated by randomly packing 800 monosaccharide molecules in a cubic box with side lengths of 54 Å using Packmol.^51^ Although pure monosaccharides are crystalline at ambient conditions, the randomly packed bulk systems used here do not crystallize during accessible MD time scales at 300 K and instead sample a metastable amorphous condensed state. Similarly, a single monosaccharide molecule was placed in a cubic box of side length 54 Å, replicating a gaseous-phase condition used as the gas-phase reference for the *H*_v_ calculation. These resulting configurations were used as the starting structures for subsequent MD simulations. All simulations were performed under periodic boundary conditions^47^ at 300 K and 1 atm (unless specified otherwise), where temperature and pressure control were achieved using the Langevin thermostat and barostat, respectively.^52,53^ A 1 ps^–1^ damping coefficient was used for Langevin dynamics. Initial energy minimization was carried out using the conjugate gradient method,^49^ followed by an isothermal-isobaric ensemble (NPT) for the bulk system and a canonical ensemble (NVT) for the single monosaccharide in the cubic box. Three independent simulations were conducted for each system to minimize statistical errors and improve the reliability of the simulation results.^23,27,54^

##### 2.1.2.1 All Atom Simulations

AA MD simulations were performed to determine the bulk properties of the monosaccharides and to generate trajectories for mapping onto the CG representation.^34,43^ AA simulations were carried out using three established FFs, Chemistry at HARvard Macromolecular Mechanics (CHARMM36),^55^ GLYCAM_06,^56^ and Optimized Potentials for Liquid Simulation-All Atom (OPLS-AA).^57^ For each FF, three independent simulations were performed using different starting configurations.

In AA MD simulations, electrostatic interactions were treated using the particle-mesh Ewald (PME) method.^58^ Nonbonded van der Waals (vdW) interactions were described using a 12-6 LJ potential, with cutoff distances of 9 Å, 10 Å, and 12 Å for OPLS-AA FF,^59,60^ GLYCAM_06 FF,^61–63^ and CHARMM FF,^55,62^ respectively. Atom pairs separated by two and three covalent bonds were excluded from nonbonded interactions using the “exclude scaled1–4” criterion. All simulations were conducted with a 1 fs time step for 20 ns unless otherwise specified, and trajectories were written every 1 ps. The last 10 ns of each trajectory were used for obtaining density and *H*_v_. The density was determined by dividing the total mass of the system by the average equilibrium volume obtained from the NPT simulations. Details of *H*_v_ analysis are provided in Section S1.2 of the SI. The average density and *H*_v_ from three FFs were used as target properties in the PSO optimization described in Section 2.1.3. The standard deviations calculated from the three FF predictions were used as the AA simulation uncertainty in the BUQ calculations, as described in Section 2.2.3.

##### 2.1.2.2 Coarse-Grained Simulations

CG MD simulations of the monosaccharides were performed to test and validate the optimized parameters obtained from the PSO procedure. For each system, three independent simulations were conducted with 800 molecules and a 5 fs time step. This timestep was selected to maintain numerical stability and avoid integration errors. Nonbonded interactions were described using a 12-6 LJ potential with a cutoff distance of 12 Å, consistent with previously developed CG models from our group. CG bead pairs directly connected by a bond were excluded from nonbonded interactions using the “exclude 1–2” criterion. Both NPT and NVT simulations were performed for 100 ns, and the last 20 ns of each trajectory were used for property analysis. Moreover, to assess the numerical stability and timestep robustness of the optimized CG FF parameters, we performed simulations using timesteps of 8 and 10 fs for the model monosaccharides.

#### 2.1.3 Parameter Development

PSO, a bio-inspired optimization algorithm for identifying optimal solutions within a defined search space, was used to optimize the CG nonbonded interaction parameters by minimizing deviations between CG and AA simulation results.^29,43,64,65,66,67,68^ Bonded parameters, including equilibrium bond lengths, angles, and dihedrals, were derived independently from the last 2 ns of the mapped AA simulation trajectories and were not included in the PSO optimization. Initial bonded force constants were assigned based on commonly used values for similar CG models.^43,44,66^

A total of eight CG parameters, corresponding to LJ interaction terms, ϵ and σ, for BB, LM, SB, and GC/BC beads, were optimized simultaneously within predefined bounds listed in Table S3. The GC and BC beads were assigned shared nonbonded parameters, reflecting their common carbon-based environments while preserving stereochemical distinctions through the bonded topology and mapping. The nonbonded parameters for the DRO and LRO beads were not optimized by PSO. Instead, the O5 ring-oxygen parameters from the CHARMM36 FF were assigned directly, because the RO bead represents a single oxygen atom.^55^

In the PSO framework, each particle represents a candidate set of CG parameters in an eight-dimensional search space. A swarm of 64 particles was initialized randomly within the predefined parameter bounds. Particle velocities were constrained by a maximum value of 0.01 in normalized parameter units along each dimension to ensure stable exploration of parameter space. PSO was performed for 300 iterations, and the swarm was reinitialized if no improvement in the global best solution was observed for 20 consecutive iterations, reducing the likelihood of stagnation in local minima. A detailed description of the PSO algorithm can be found in the CG water model development work by Bejagam et al.^64^

The CG MD simulations in the PSO algorithm were carried out using the NAMD 2.14.^49^ For each candidate parameter set, NPT simulations of 800 CG molecules were performed at 300 K and 1 atm for 5 ns using a 5 fs timestep. The last 2 ns of each simulation were used to compute target properties, density and *H*_v_, for each of the three model monosaccharides. The computed properties, *Y_i_* ^CG^, were then compared against AA reference values, *Y_i_* ^target^, obtained from AA simulations of the same bulk systems. The six target properties were combined into a single objective function defined as a weighted sum of relative errors

2 $$\begin{array}{ll} E=\sum_{i=1}^{6} & \omega_{i}\left|\frac{Y_{i}^{CG}-Y_{i}^{target}}{Y_{i}^{target}}\right| \end{array}$$

Equal weights, ω*_i_* = 1, were assigned to all properties so that each property and each monosaccharide contributed equally to the optimization. This formulation drove PSO to minimize relative deviations across all target properties simultaneously, thereby reducing bias toward any single property or molecule. The objective-function value was used by the PSO algorithm to update each particle’s velocity and position based on its individual best solution and the global best solution identified by the swarm.

Following nonbonded parameter optimization, CG simulations were performed using the protocol described in Section 2.1.2.2, and the resulting bonded distributions were compared against the corresponding AA-mapped distributions. Minor adjustments to the bonded force constants were then applied to improve agreement in bond length, angle, and dihedral distributions. This iterative refinement ensured that the CG model reproduced local structural features while remaining consistent with the optimized nonbonded interactions. Variations in bonded force constants had a negligible impact on the target thermodynamic properties, density and *H*_v_, consistent with the expectation that these condensed-phase properties are governed primarily by nonbonded interactions.^45^

As described in Section 2.1.1, the charge-neutral LJ representation implicitly combines several physical contributions, and therefore, different combinations of LJ parameters may reproduce the target properties with comparable accuracy through compensating effects. PSO identified a single global-best parameter set, which was selected as the FF parameterization because it produced the lowest weighted objective-function value across the six calibration targets. However, this point estimate is not assumed to be uniquely determined by the available data, and PSO alone does not quantify how uncertainties in the target data and surrogate-model predictions, along with correlations among the parameters, affect the range of statistically admissible FF parameter sets. BUQ was therefore introduced as a second stage to identify alternative parameter combinations that remain statistically consistent with the target data and to quantify parameter uncertainty, correlations, and non-uniqueness.

### 2.2 Uncertainty Quantification and Robustness Assessment

BUQ was introduced as a post-optimization analysis and used to quantify uncertainty in CG FF parameters and predicted properties by estimating posterior parameter distributions conditioned on the AA-derived target data. This approach enables identification of parameter regions that are statistically consistent with the target properties while accounting for uncertainty in both the reference data and surrogate model predictions. The BUQ workflow adopted in this work follows a structured, multi-step procedure described in the following sections.^36,69^

#### 2.2.1 Sobol Sequence-Based Dataset Generation

Starting from the PSO-optimized parameter set, we generated a local parameter-space dataset for surrogate-model training, sensitivity analysis, and Bayesian inference. A Sobol sequence-based perturbation scheme was used to generate 5000 new parameter sets, each obtained by perturbing the PSO-optimized CG parameters by up to ±5% according to eqs 3 and 4 ^36,70,71^

3 $$\theta_{\min}=\theta \left(1-\delta \right);\theta_{\max}=\theta \left(1+\delta \right),\delta =5\%$$

4 $$\theta_{i}=\theta_{\min}+\left(\theta_{\max}-\theta_{\min}\right)SP_{i},i\in \left\{1,2,...,N\right\}$$

where θ is the parameter set, δ is the perturbation percent, and SP*_i_* is the *i*th point in the Sobol sequence. Using these 5000 parameter sets, we performed MD simulations of the model monosaccharides to obtain density and *H*_v_ (5000 sets × 3 systems × 2 properties = 30,000 MD simulations). These data served as the training and testing datasets for the GPR model, explained in the following section.^72^ We incorporated the MD results obtained into eq 5 and obtained the Sobol sensitivity indices. Sobol sensitivity indices analyze how much each input parameter contributes to the variability in an output property.^69,73^

5 $$\mathrm{SI}=V\left[E\left(Y|X_{i}\right)\right]/V\left(Y\right)$$

where *Y* is the output property, *X_i_* is the input parameter, *V*(*Y*) is the total variance in the output property across all 5000 data, and *V*[*E*(*Y*|*X_i_*)] is the variance in *Y* caused by changes in *X_i_* alone. The Sobol index quantifies the fraction of the total variance *V*(*Y*) explained by a specific parameter. A first-order index measures the direct effect of a single parameter on the output property, whereas a total-order index measures the combined effect of a parameter, including its direct contribution and its interactions with other parameters. The variance of the output property arises from both individual parameter effects and interaction terms between two or more parameters. Using eq 5, the effect of higher-order interactions can be assessed by comparing first and total-order indices. The Sobol sensitivity indices ensure that we focus on the significant parameters and parameter interactions affecting particular properties.

#### 2.2.2 Gaussian Process Regression Model

Gaussian Process Regression (GPR) is a nonparametric machine-learning approach that, unlike conventional regression models, provides a predictive distribution rather than only a point estimate at each input.^72^ This makes GPR naturally compatible with Bayesian inference because both the predicted mean and the surrogate-model uncertainty can be incorporated into the likelihood function. The CG MD results generated from the 5000 Sobol-sampled parameter sets were used to train GPR surrogate models for the target properties. These surrogates replaced direct MD simulations during Bayesian inference, enabling efficient evaluation of the likelihood across parameter space. For training and validation, the dataset was split into 4000 training samples and 1000 test samples. Multiple kernel functions were evaluated, and the best-performing kernel was selected for each target property based on predictive accuracy on the test set. After kernel selection, the final surrogate model for each property was trained using the full set of 5000 Sobol-sampled data points. Additional details of the GPR implementation, kernel selection, and model-performance metrics are provided in Section S3 of the SI.

#### 2.2.3 Bayesian Inference and Posterior Characterization

Bayesian inference was used to evaluate the relative plausibility of parameter combinations within the locally sampled region surrounding the PSO solution.^74^ Unlike PSO, this procedure did not minimize a new objective function. Instead, we used Bayes’ theorem to estimate the probability distributions of these parameters based on how well the model’s predictions align with target observed data (*Y*_obs_).^75,76^

6 $$p\left({\theta |Y}_{obs},M\right)=\frac{p\left(Y_{obs}|\theta ,M\right)\cdot p\left(\theta |M\right)}{p\left(Y_{obs}|M\right)}$$

Equation 6 defined the posterior probability distribution, *p*(θ|*Y*_obs_, *M*), which represents the probability of a given set of CG FF parameters (θ), given the observed AA data, *Y*_obs_, and the surrogate model, *M*. In the present work, this distribution quantifies which regions of the parameter space yield model predictions (*Y*_pred_) that are consistent with the target thermodynamic properties within their associated uncertainties (ε in eq 7). The posterior probability distribution may be summarized by its maximum a posteriori (MAP) parameter set, corresponding to the parameter combination with the highest posterior probability under the specified prior, likelihood, and uncertainty assumptions. In this work, the MAP set was used as a statistical summary of the posterior and as a reference point for the subsequent robustness analysis. It was not treated as a second optimized FF parameter set.

The likelihood term, *p*(*Y*_obs_|θ, *M*), quantifies how well the model’s predictions (*Y*_pred_) align with the AA observations (*Y*_obs_). Assuming normally distributed deviations, the relationship between prediction and observation is expressed as eq 7.

7 $$Y_{\mathrm{obs}}=Y_{\mathrm{pred}}+\varepsilon$$

where *Y*_pred_ is the predicted value from the model, *M*, and ε is the total error in measurement, which includes measurement error from AA simulations (ε_D_) and modeling error of GPR (ε_SM_).

8 $$\varepsilon =\varepsilon_{D}+\varepsilon_{\mathrm{SM}}$$

Under this assumption, parameter sets that produce predictions closer to the target values are assigned a higher likelihood. A uniform prior distribution, *p*(θ|*M*), was employed within the same bounds used for Sobol sequence sampling. This choice avoids introducing bias towards any specific region of the parameter space, allowing the posterior to be primarily informed by the data through the likelihood function. *p*(*Y*_obs_|*M*) is the normalization factor, which ensures probabilities sum to 1, which is assumed to be constant. The resulting posterior distribution therefore provides a probabilistic description of the CG parameter space, identifying not only a single optimal parameter set but a range of parameter combinations that are consistent with the AA data.

The integration of the GPR model, the surrogate for MD simulations, and PEUQSE,^75,77^ a Bayesian tool designed for parameter estimation, enabled us to extensively sample the parameter sets and obtain a posterior distribution, which reflects the uncertainty about the exact parameters obtained from PSO. The likelihood was constructed as a multivariate Gaussian. Ensemble Slice Sampling (ESS) was used with 32 walkers and 2 × 10^6^ samples to achieve adequate parameter-space exploration.^36^ Integrated autocorrelation time (ACT) was used as the convergence diagnostic. From the converged posterior distribution, 100 parameter sets were randomly sampled after convergence and CG MD simulations were conducted on these using the protocol described in Section 2.1.2.2 for further validation. The resulting property predictions were compared with those obtained from the GPR surrogate models to assess surrogate accuracy within the parameter region most relevant to Bayesian inference. More details can be found in Section S6 of the SI.

#### 2.2.4 Parameter Robustness Assessment

To evaluate the robustness of the optimized CG parameters, we performed an additional analysis following BUQ-based posterior characterization. The objective was to identify parameter ranges over which the CG model continues to reproduce output properties within acceptable statistical uncertainty. Starting from the MAP estimates obtained from BUQ, the local parameter space around the MAP values was systematically sampled by generating random parameter perturbations. For each generated parameter set, the output properties were predicted using the trained surrogate models. A parameter range was classified as robust if all property predictions obtained from parameter variations within that range remained within the 95% confidence intervals of the target values, approximated as μ ± 2σ, where μ and σ denote the mean and standard deviation, respectively. The perturbation magnitude was iteratively adjusted until the sampled parameter sets satisfied this criterion. This procedure enabled identification of parameter regions over which the CG model maintains predictive accuracy and provided guidance on which parameters can be varied without compromising model performance.

### 2.3 Transferability Evaluation

To assess the transferability of the optimized CG parameters, MD simulations were performed for the validation monosaccharides shown in Figure S1. The CG parameters obtained from PSO were applied without further modification. All simulations employed the same setup described in Section 2.1.2. The density and *H*_v_ of the validation monosaccharides were calculated to assess whether parameters optimized using the model monosaccharides could reliably reproduce thermodynamic properties for chemically similar systems not included in the optimization procedure.

### 2.4 Validation against Independent Properties

To evaluate the predictive capability of the developed CG FF parameters beyond the properties used during optimization, we assessed their ability to reproduce atomistic surface energy data and experimental glass transition temperatures for 12 monosaccharides (model and validation monosaccharides). These properties were not used as targets during PSO and therefore provide an independent test of model performance. The methods of analysis are discussed in the following sections.

#### 2.4.1 Surface Energy

To further evaluate the predictive capability of the CG FF for interfacial properties, we calculated surface energies using a well-established slab-based approach from MD simulations.^36,78^ Physically, surface energy is the measure of the energy change associated with breaking a bulk material and exposing a surface.^79^ In MD simulations, this can be achieved by computing the difference in potential energy between the slab and bulk systems. We first extracted the equilibrated configurations from NPT simulations, discussed in Section 2.1.2. These equilibrated configurations were then simulated in the NVT ensemble to obtain reference bulk energies (*U*_bulk_) according to eq 1.

To construct a vacuum-carbohydrate interface, the equilibrated bulk configurations were expanded along the *z*-direction by ∼five times the *x* or *y* axis, to introduce a vacuum region while preserving the *x* and *y* dimensions, as shown in Figure 3. This created a slab geometry with two vacuum-carbohydrate interfaces. These slab systems were simulated for 200 ns under NVT conditions, and the potential energy (*U*_slab_) was calculated from the last 50 ns. Surface energies were computed from the difference between the average potential energies of the slab and bulk systems, normalized by twice the interfacial area, according to eq 9.

9 $$\gamma \approx \frac{U_{slab}-U_{bulk}}{2A}$$

**Figure 3:**
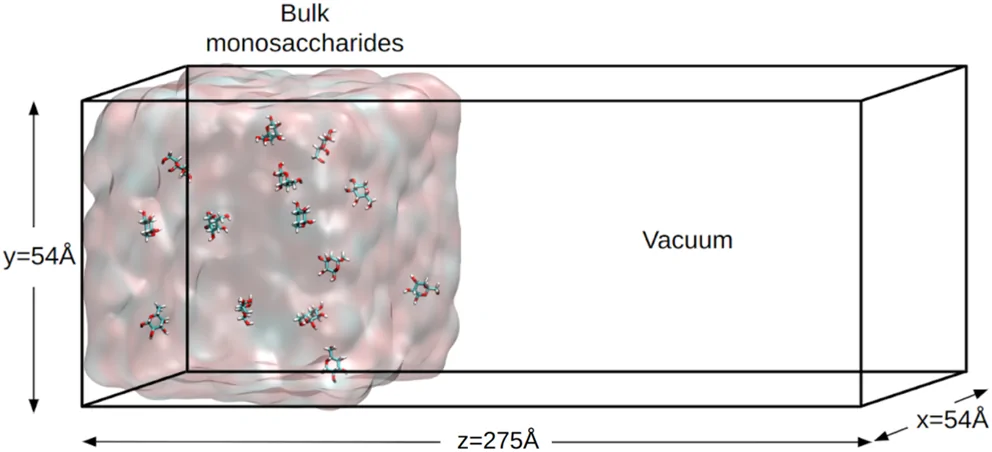
Schematic representation of the simulation setup for the MD simulation to obtain surface energy. Monosaccharides are shown in the quick-surf and licorice representations according to VMD.

where *A* is the cross-sectional area in the *xy* plane. This procedure was applied to both atomistic (AA) and CG models. CG predictions were evaluated against corresponding AA simulation results.

#### 2.4.2 Glass Transition Temperature (*T*_g_)

We evaluated the developed CG FF across a range of temperatures by assessing its ability to reproduce the glass transition temperature (*T*_g_) of monosaccharide systems. For bulk amorphous monosaccharides, *T*_g_ represents a transition, where the molecular structure of individual monosaccharides remains unchanged, while the mobility of the amorphous bulk changes dramatically as molecular relaxation times become comparable to the observation time scale.^80–83^ Below *T*_g_, the disordered monosaccharides exist in a kinetically trapped glassy state in which translational and cooperative intermolecular rearrangements are severely restricted. Above *T*_g_, free volume and molecular mobility increase, allowing structural relaxation and diffusion within the amorphous matrix.^81,84,85^ Consequently, the material transitions from a rigid glassy solid to a supercooled liquid state without undergoing a first-order phase transition. Such glass-transition behavior has been experimentally observed for amorphous monosaccharides.^83^ In MD simulations, *T*_g_ is estimated from the change in slope of temperature-dependent properties such as specific volume or density across the transition region. Because the present CG model is parameterized using condensed-phase bulk monosaccharide systems, reproducing *T*_g_ provides an additional validation of the model’s ability to capture the collective thermodynamic behavior and intermolecular packing of amorphous carbohydrate phases.

In aqueous environments, these monosaccharides exist as mixtures of α and β anomers in equilibrium. However, since the present *T*_g_ calculations were conducted in the absence of solvent, separate simulations were performed for the pure α and pure β forms (100% α or 100% β systems). For each monosaccharide, bulk systems were generated by randomly packing 1000 monosaccharide molecules in a cubic box with side lengths of 60 Å using Packmol.^51^ All simulations were performed in the NPT ensemble using the same simulation protocol described in Section 2.1.2 of the main manuscript. Following the method by Simperler et al., we carried out temperature-dependent simulations over a range of 520 to 210 K in decrements of 10 K, simulating at each temperature for 100 ns.^86^ To imitate a continuous cooling process and maintain structural continuity, the final configuration and box dimensions obtained at each temperature were used as the initial conditions for the subsequent lower-temperature simulation. At each temperature, the equilibrium volume was obtained from the last 10 ns of the trajectory, and the corresponding specific volume (volume per unit mass) was calculated. The variation of specific volume with temperature was then used to determine *T*_g_. This approach relies on the characteristic change in slope of the specific volume and temperature relationship. The data were divided using a cutoff temperature value, and linear fits were applied to each region. *T*_g_ was then estimated as the intersection of these two linear fits. The quality of the linear regressions was evaluated using the coefficient of determination (*R* ^2^), and only fits with mean *R* ^2^ ≥ 0.995 were considered. When multiple valid fitting ranges satisfied this criterion, the corresponding *T*_g_ values were averaged.

## 3 Results and Discussion

### 3.1 Bulk Thermophysical Properties

The average density and *H*_v_ of the monosaccharides obtained from three independent simulations per FF were consistent with each other, as shown in Table S2, supporting their reliability as benchmarks for CG parameterization. The final optimized nonbonded CG FF parameters obtained through PSO are reported in Table S3, and bonded, angle, and dihedral angle parameters are reported in Tables S4–S6, respectively.

Table 1 reports the density and *H*_v_ from CG MD simulations alongside the AA-derived target values. The error is reported as a percent difference relative to the AA reference. The CG runs showed low variability across independent replicates, indicating stable and reproducible sampling. Density errors remained below 2.5% and *H*_v_ errors below 3.5% for all three solutes, demonstrating that the optimized nonbonded parameters reproduce the target bulk properties with good accuracy. The larger AA standard deviations reflect spread across the three FFs rather than within-replicate noise.

**Table 1:** Density and *H*_v_ of Model Monosaccharides at 300 K^a^ ^,^ ^b^

	density (g/cm^3^)			heat of vaporization (kcal/mol)		
monosaccharides	CG	AA^a^	%error	CG	AA^a^	%error
α-d-glucose	1.467 ± 0.002	1.435 ± 0.046	2.23	29.13 ± 0.08	30.01 ± 4.63	2.93
β-d-xylose	1.407 ± 0.001	1.423 ± 0.046	1.12	25.33 ± 0.02	26.20 ± 1.77	3.32
α-l-rhamnose	1.298 ± 0.001	1.331 ± 0.035	2.48	26.13 ± 0.14	26.18 ± 2.64	0.19

a AA indicates the average from three FFs, where three independent simulations were performed with each FF.

b Results from CG MD simulations are compared with average values obtained from three AA FFs.

Density and *H*_v_ distributions obtained from simulations conducted with 8 and 10 fs timesteps show good agreement with reference AA data, as shown in Figure S2. This indicates that the optimized CG FF yields timestep-independent thermodynamic properties within this timestep range.

### 3.2 Structural Features

To assess whether the optimized CG model preserves the local structure of the mapped AA reference systems, bond length, bond angle, dihedral angle, and radial distribution function (RDF) distributions were computed from the last 20 ns of the CG MD trajectories for each model monosaccharide. These comparisons, presented in Figures 4 to 8, evaluate both the bonded interactions that define pyranose ring geometry and the intermolecular packing behavior produced by the optimized nonbonded parameters.

**Figure 4:**
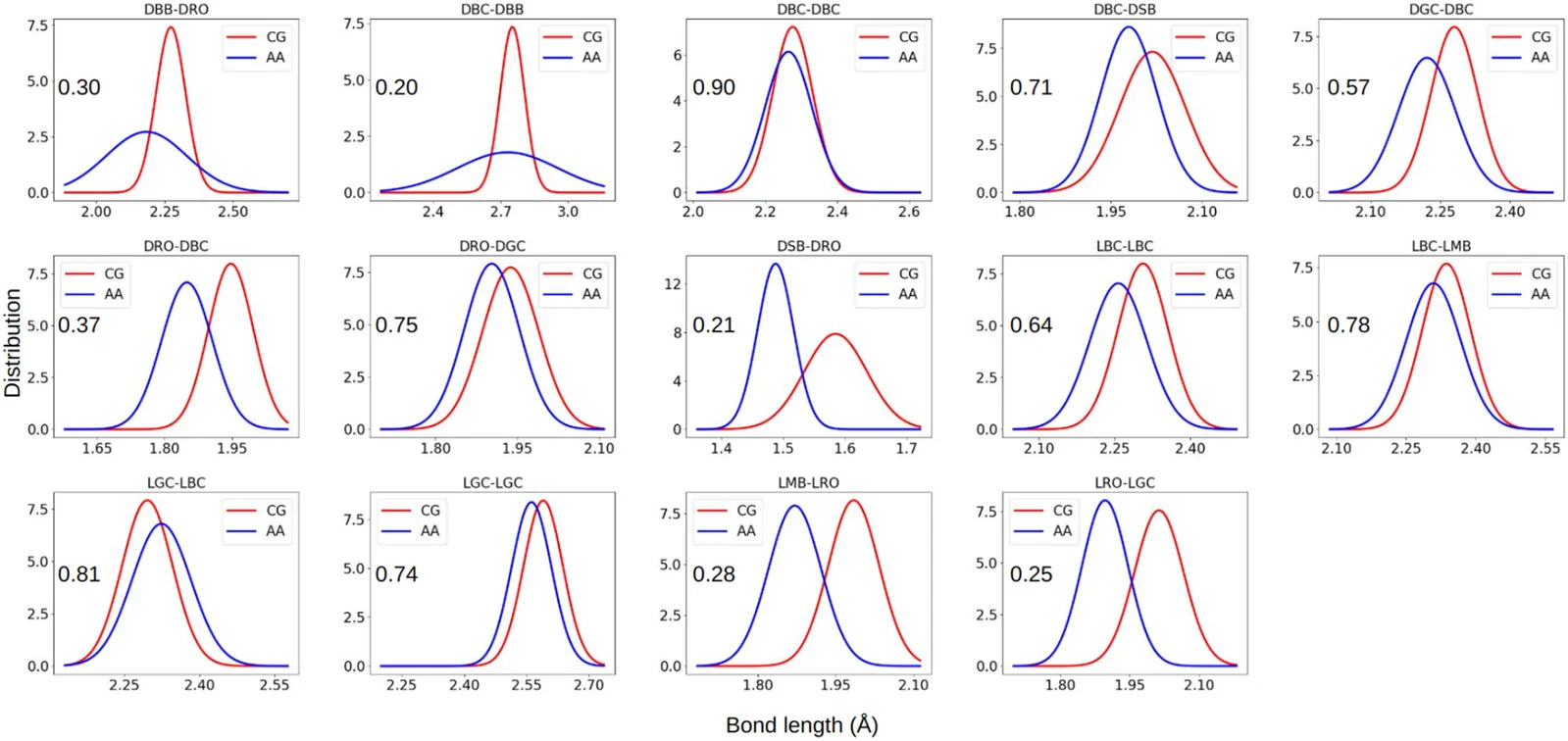
Bond length distributions for the model monosaccharides from CG MD simulations and mapped AA trajectories. Annotated values on the plots show an overlap coefficient (OC). CG bead names are defined in Table S1.

Agreement between CG and AA distributions was quantified using the overlap coefficient (OC), which measures the shared area between two probability density functions. OC values range from 0, indicating no overlap, to 1, indicating perfect overlap,^87^ and have been used across many fields to compare distributions and assess model agreement.^88–90^ Distributions with OC ≥ 0.5 were considered to show reasonable agreement; those with OC < 0.5 were examined further to determine whether the discrepancy arose from peak shifts, differences in distribution width, or reduced representation of fine structural features at CG resolution.

The CG model reproduces the bond length distributions with moderate to high accuracy, with OC values ranging from 0.20 to 0.90 (Figure 4). Most bond types show reasonable agreement with the mapped AA references, particularly DBC-DBC, DBC-DSB, DGC-DBC, DRO–DGC, LBC-LBC, LGC-LBC, LGC-LGC, and LBC-LMB, where the CG and AA distributions have similar peak positions and substantial overlap. The lower-overlap cases arise from two main sources. For α-d-glucose, the DBB-DRO and DBC-DBB distributions show OC values of 0.30 and 0.20, respectively, primarily because the CG distributions are narrower than the mapped AA distributions. The DRO–DBC distribution shows an OC of 0.37, with the CG distribution shifted to larger bond lengths. For β-d-xylose, the DSB-DRO distribution shows a pronounced shift to larger bond lengths, resulting in an OC of 0.21. Similarly, the LMB-LRO and LRO-LGC distributions in α-l-rhamnose are shifted to larger bond lengths, giving OC values of 0.28 and 0.25, respectively. These deviations suggest that while the CG model captures the dominant bond-length features for most bead pairs, selected bonds involving smaller or more chemically specific beads are more sensitive to the reduced representation and local bonded constraints.

The bond angle distributions show similar behavior (Figure 5), with OC values ranging from 0.06 to 0.90. Most angle types show reasonable to strong overlap with the mapped AA references, including DBC-DBB-DRO, DBC-DBC-DBB, DBC-DBC-DBC, DBC-DSB-DRO, DGC-DBC-DBC, DRO–DBC-DBC, DRO–DGC-DBC, DSB-DRO–DBC, LBC-LBC-LMB, and LBC-LMB-LRO. These results indicate that the optimized bonded parameters preserve the dominant angular preferences for most bead environments. The lower-overlap cases are concentrated in selected angles rather than distributed across the entire bonded network. For α-d-glucose, the DBB-DRO–DGC angle has an OC of 0.47, mainly because the CG distribution is narrower than the mapped AA distribution. For β-d-xylose, the DBC-DBC-DSB angle shows a shift to larger values, giving an OC of 0.42. For α-l-rhamnose, the LGC-LBC-LBC, LGC-LGC-LBC, LMB-LRO-LGC, and LRO-LGC-LGC angles show reduced overlap, with OC values of 0.42, 0.31, 0.44, and 0.06, respectively. These deviations indicate that the CG model preserves the major angular features of the pyranose geometry but is less accurate for selected local environments, especially those involving bead types associated with xylose and rhamnose.

**Figure 5:**
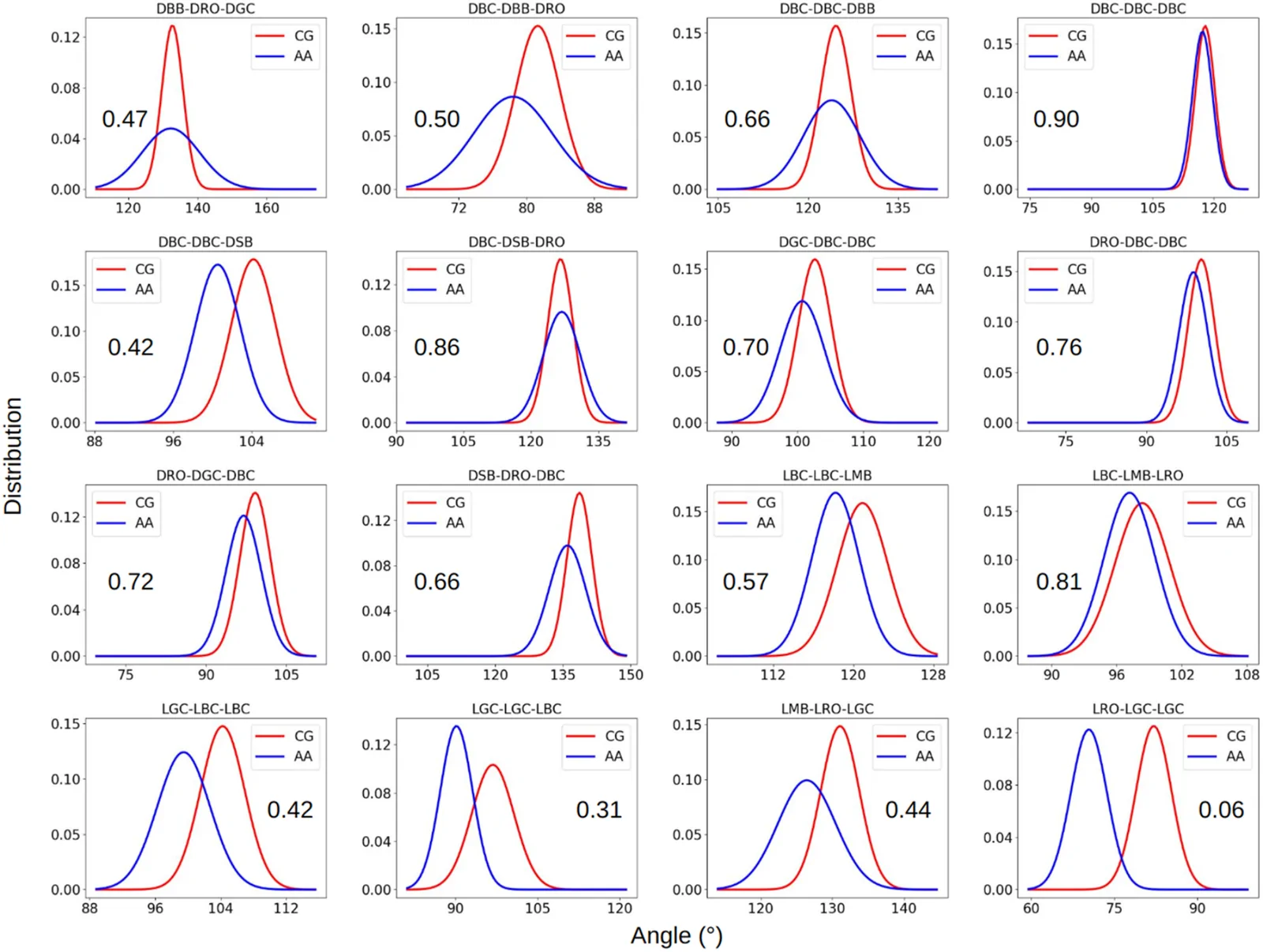
Bond angle distributions for the model monosaccharides from CG MD simulations and mapped AA trajectories. Annotated values on the plots show an overlap coefficient (OC). CG bead names are defined in Table S1.

For the d-enantiomer model monosaccharides, most dihedral distributions show reasonable to strong agreement with the mapped AA references, with OC values generally above 0.5 (Figure 6). The CG model captures the major conformational states associated with the pyranose ring, including several distributions with substantial overlap, such as BC-BC-BC-BC, BC-BC-BC-SB, RO-BC-BC-BC, RO-GC-BC-BC, and SB-RO-BC-BC. The main exception is the BC-SB-RO-BC dihedral in β-d-xylose, which has an OC of 0.37 because the CG distribution is shifted relative to the mapped AA distribution. This deviation likely reflects the greater sensitivity of the smaller SB bead environment to local bonded constraints and packing effects.

**Figure 6:**
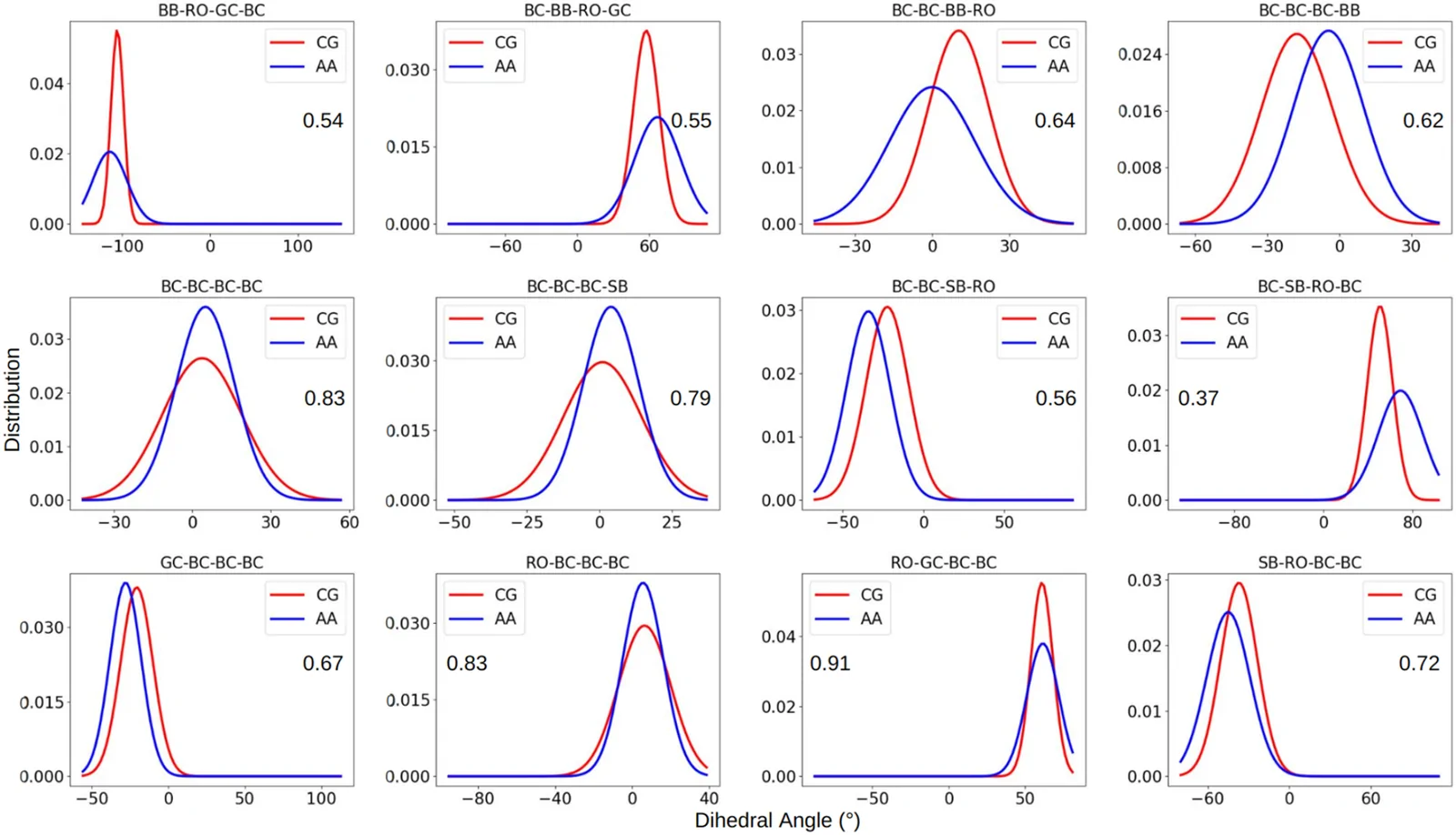
Dihedral angle distributions for the d-enantiomer model monosaccharides α-d-glucose and β-d-xylose from CG MD simulations and mapped AA trajectories. Annotated values on the plots show an overlap coefficient (OC). CG bead names are defined in Table S1.

For α-l-rhamnose, the CG model reproduces most mapped AA dihedral distributions with reasonable agreement (Figure 7). Four of the six dihedral distributions have OC values above 0.5, indicating that the dominant conformational preferences of the l-enantiomer pyranose ring are largely preserved. The strongest deviations occur for the MB-RO-GC-GC and RO-GC-GC-BC dihedrals, with OC values of 0.25 and 0.23, respectively. These reduced overlaps arise from shifts in the CG probability distributions relative to the mapped AA references, with MB-RO-GC-GC shifted to lower dihedral angles and RO-GC-GC-BC shifted to higher dihedral angles. The lower overlap observed for selected β-d-xylose and α-l-rhamnose bonded distributions may be associated with the smaller DSB and LMB beads used for xylose and rhamnose, respectively. Their reduced effective size can increase sensitivity to local packing and bonded constraints, leading to shifts in the CG distributions relative to the mapped AA references.

**Figure 7:**
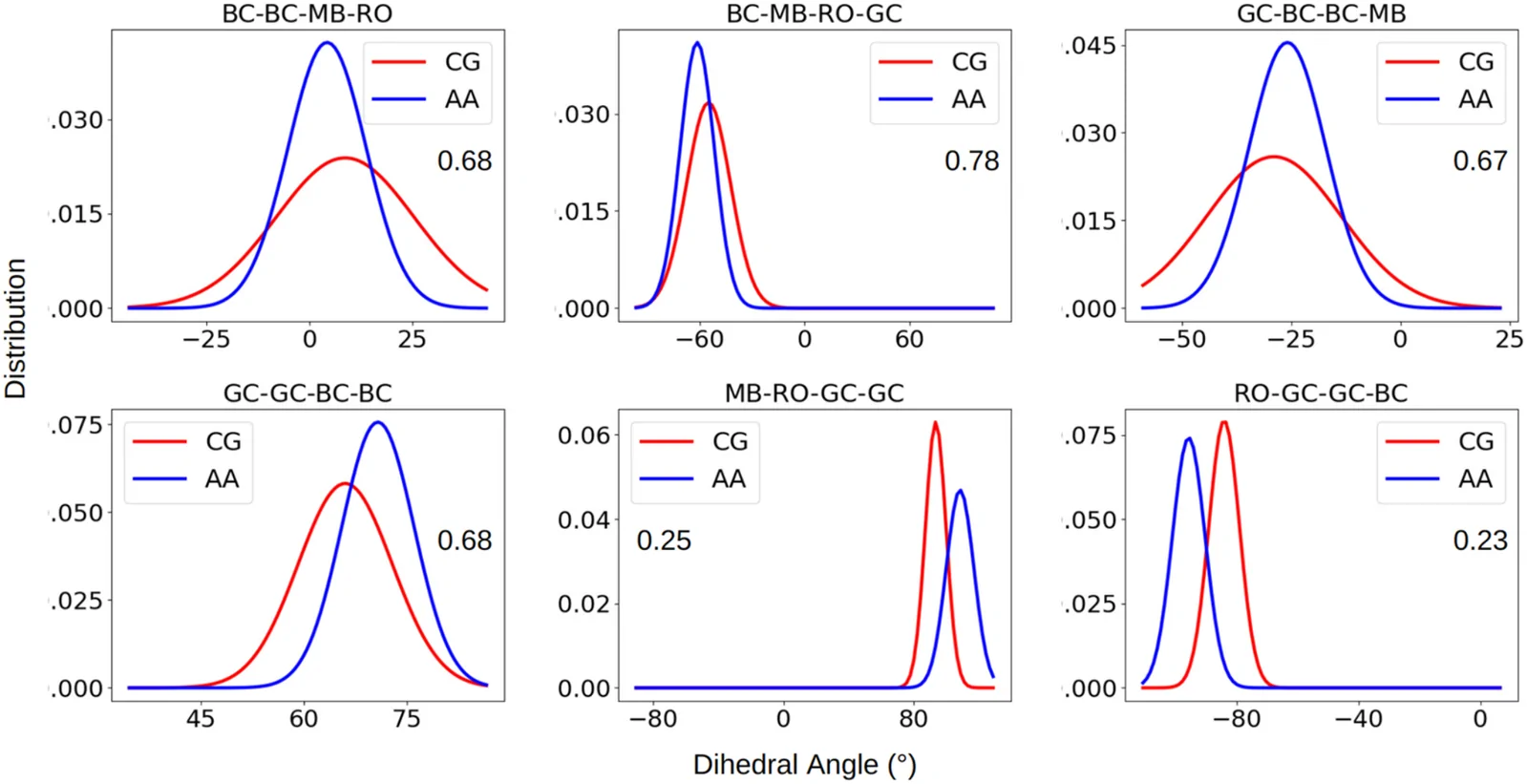
Dihedral angle distributions for the l-enantiomer model monosaccharide α-l-rhamnose from CG MD simulations and mapped AA trajectories. Annotated values on the plots show an overlap coefficient (OC). CG bead names are defined in Table S1.

The CG model captures the principal intermolecular packing features observed in the mapped AA RDFs, including the onset of the first coordination shell and the approach to bulk-like behavior at larger distances (Figure 8). For CG bead pairs except DRO–DRO and LRO-LRO, CG RDFs exhibit sharper first-shell features than the mapped AA references. This behavior is consistent with stronger effective bead–bead attractions introduced through the optimized nonbonded parameters. For DBC-DBC, DGC-DGC, LBC-LBC, and LGC-LGC, the CG first peaks also lie at slightly larger separations than the AA references, consistent with the larger optimized σ values for these bead types. The DRO–DRO and LRO-LRO RDFs show comparatively good agreement in the broad shape of the first coordination shell, suggesting that ring-oxygen interactions are reasonably preserved despite the charge-neutral CG representation. Secondary-shell features are less consistently reproduced, which is expected for a reduced-resolution model that averages over atomistic packing correlations. Despite these deviations, the CG model retains the dominant intermolecular organization needed to describe bulk packing behavior in the model monosaccharides.

**Figure 8:**
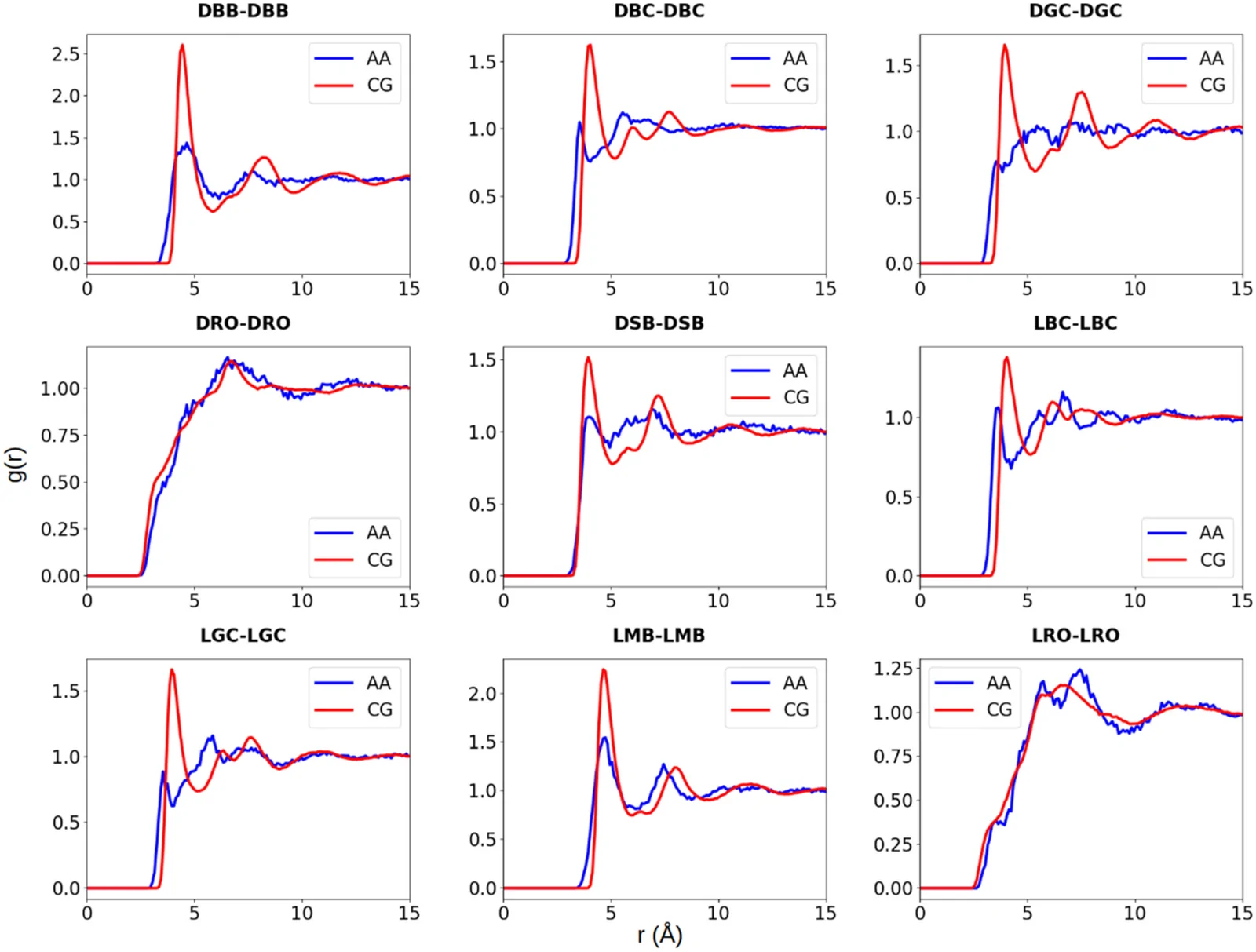
Radial distribution functions for the model monosaccharides from CG MD simulations and mapped AA trajectories. CG bead names are defined in Table S1.

Taken together, the bonded and RDF analyses show that the CG model reproduces the primary structural features of the mapped AA systems while retaining a transferable bead representation. Lower OC values are concentrated in specific bead environments, particularly those involving the smaller DSB and LMB beads in xylose and rhamnose, whose reduced effective size likely increases sensitivity to local packing and bonded constraints. These effects produce the observed peak shifts and width differences. Such discrepancies are expected for a transferable CG model optimized jointly for bulk properties and structural fidelity. The present parameter set therefore provides a balanced transferable representation for CG modeling of pyranose monosaccharides.

### 3.3 Property Trends in CG MD Training Data

To evaluate the sensitivity of the target bulk properties, density and *H*_v_, with respect to variations in input parameters, raincloud plots were constructed from the CG MD results obtained using 5000 Sobol-sampled parameter sets (Figure 9). Each raincloud plot combines a kernel density estimate, the underlying data points, and a box plot showing the median and interquartile range, providing a compact representation of the predicted property distributions.

**Figure 9:**
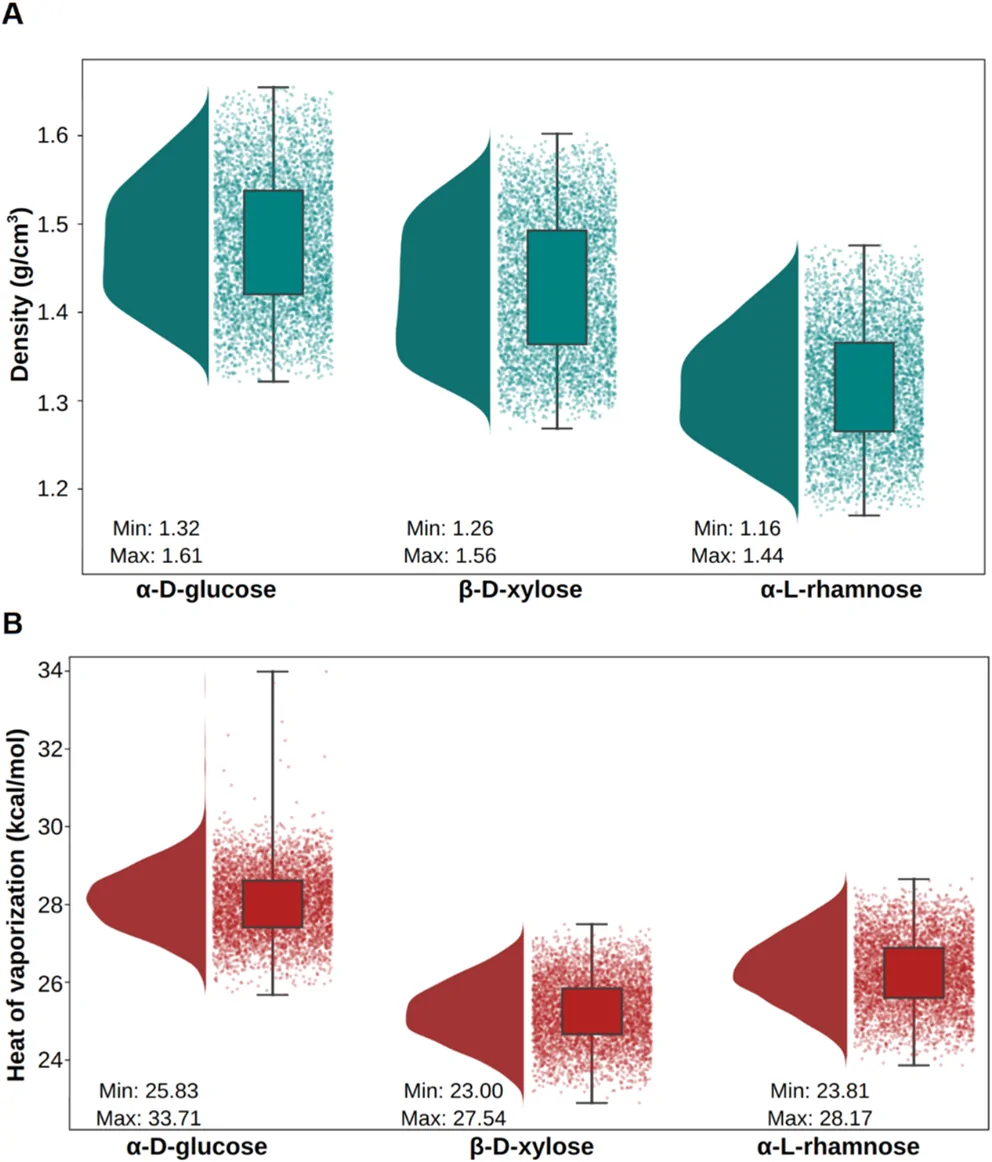
Raincloud plots showing the variation in (A) density and (B) *H*_v_, calculated from CG MD simulations using Sobol-perturbed input parameters. Min and Max indicate the minimum and maximum values predicted across the 5000 parameter sets.

The density distributions for the three monosaccharides are relatively narrow, with coefficients of variation (CoV) ranging from 4.11 to 4.96% (Table S7), suggesting that density is only weakly sensitive to parameter perturbations within the selected parameter bounds. In contrast, the *H*_v_ distributions are broader, reflecting a stronger dependence on the nonbonded interaction parameters, particularly ϵ, consistent with *H*_v_ being a direct probe of intermolecular cohesive energy.^45^ Among the three model monosaccharides, α-d-glucose shows the widest *H*_v_ distribution, ranging from 25.83 to 33.71 kcal/mol, compared with 23.00 to 27.54 kcal/mol for β-d-xylose and 23.81 to 28.17 kcal/mol for α-l-rhamnose. This indicates that the sensitivity of *H*_v_ to the underlying FF parameters is molecule-dependent, which is expected because these monosaccharides contain both unique beads and several common beads. Overall, the observed variation confirms that the selected parameter bounds and Sobol sampling strategy span a meaningful region of parameter space. The resulting dataset, therefore, provides sufficient diversity for training the GPR surrogate models used in the subsequent BUQ analysis.

### 3.4 Gaussian Process Regression (GPR) Model

Table S7 reports the R^2^ values and best-performing kernels for each property, along with the coefficient of variation (CoV) computed within the 5% perturbation range around the PSO-optimized parameter set. The CoV remained below 5% in all cases, indicating controlled property variation within the sampled parameter bounds. The GPR models achieved high predictive accuracy across all properties, with *R* ^2^ values ranging from approximately 0.94 to 0.99. The parity plots in Figure S3 further confirm strong agreement between MD-computed and GPR-predicted values. For density, the data points are tightly clustered around the parity line across three model monosaccharides. While the *H*_v_ models exhibit slightly greater scatter and a small number of outliers conserved for α-d-glucose, they maintain excellent overall agreement. These results indicate that the GPR surrogates accurately capture the underlying parameter-property relationships and are suitable for subsequent uncertainty quantification analyses.

The trained GPR models were then used to evaluate the sensitivity of monosaccharide properties to the input parameters through Sobol sensitivity indices (Figure S4). As shown in Figure 2, each model monosaccharide is represented by six CG beads, four of which, DGC, DBC, LGC, and LBC, are common across the monosaccharides and share the parameters ϵ_1_ and σ_1_. Because these common beads constitute a major fraction of each molecular representation, their parameters dominate the sensitivity trends. Specifically, σ_1_ makes the largest contribution to density across all three model monosaccharides, whereas ϵ_1_ dominates *H*_v_. The ϵ parameters associated with the unique DBB, LMB, and DSB beads, corresponding to ϵ_4_ in α-d-glucose, ϵ_3_ in α-l-rhamnose, and ϵ_2_ in β-d-xylose, respectively, contribute primarily to *H*_v_. This separation reflects the underlying physical roles of the nonbonded parameters, ϵ controls interaction strength and cohesive energy, while σ controls bead size and excluded-volume packing.^91^

### 3.5 Bayesian Uncertainty Quantification Analysis

Uncertainty quantification is rarely reported in CG FF development, where optimized parameters are often presented as single deterministic values without assessing parameter identifiability, posterior uncertainty, or uncertainty propagation to predicted properties.^36^ This limitation is particularly important for CG carbohydrate models because their structural diversity, strong hydration effects, and limited benchmark data make parameter transferability and robustness difficult to establish using point estimates alone. To address this gap, we applied BUQ to characterize the posterior distributions of CG FF parameters and the resulting uncertainty in predicted density and *H*_v_.

#### 3.5.1 Posterior Distributions for Parameters and Properties

The ESS algorithm was used to explore the CG FF parameter space and identify parameter sets that are statistically consistent with the target property data. BUQ provides a maximum a posteriori (MAP) parameter set, representing the most probable parameter combination given the target data, prior assumptions, and uncertainty model,^36,69^ and full posterior distributions over the admissible parameter space. These posterior distributions quantify uncertainty around the model and capture correlations among parameters, between parameters and properties, and among predicted properties.

The integrated autocorrelation time (ACT) analysis for the combined parameters, shown in Figure S5, indicates that the sampler achieved adequate convergence over the sampled parameter space. Figure 10 shows corner plots of the posterior distributions for α-d-glucose, β-d-xylose, and α-l-rhamnose. Additional details on corner-plot interpretation are provided in Section S5 of the SI.

**Figure 10:**
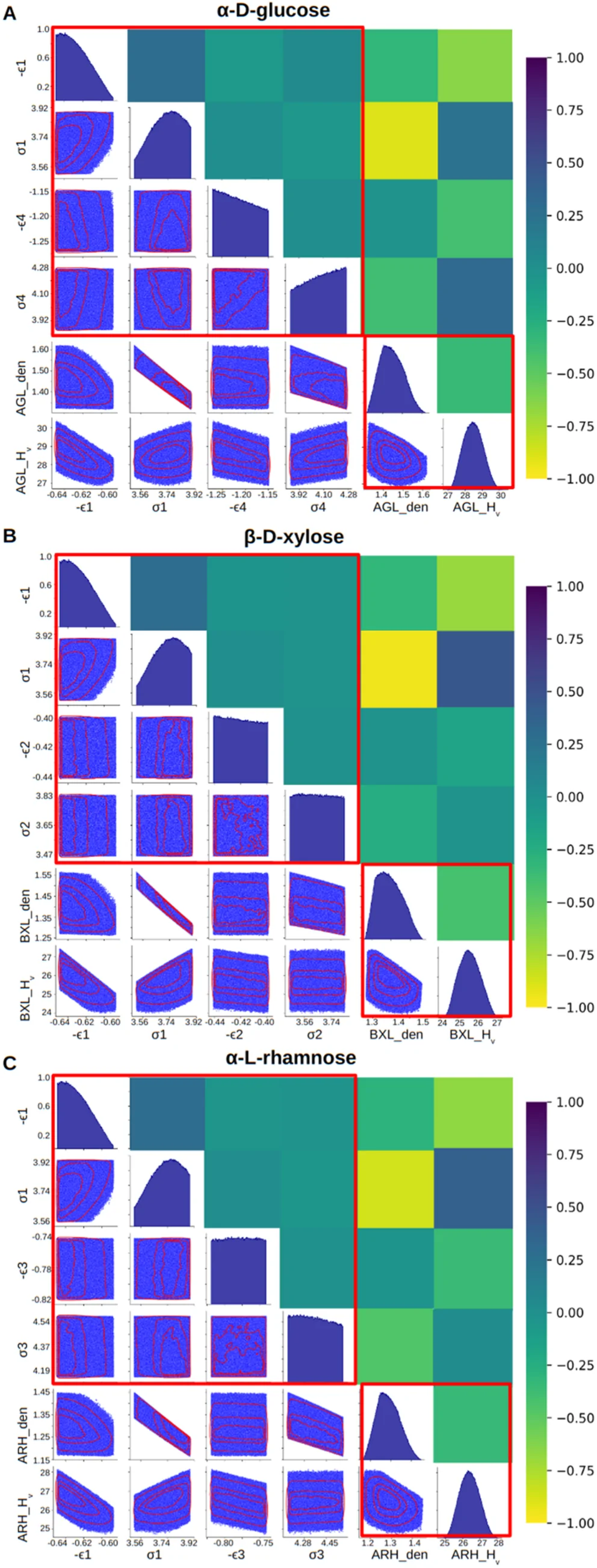
Corner plot for α-d-glucose density (AGL_den) and *H*_v_ (AGL_H_v_), β-d-xylose density (BXL_den) and *H*_v_ (BXL_H_v_), and α-l-rhamnose density (ARH_den) and *H*_v_ (ARH_H_v_), and the parameters of the constituent CG beads. The red internal border separates parameters from properties: the upper-left region, 4 × 4, shows parameter-parameter correlations, the bottom-right region, 2 × 2, shows property-property correlations, and the bottom-left region, 2 × 4, shows property-parameter correlations. Note, *E*_min_ = −ϵ values are reported here.

Across all three model monosaccharides, density shows a clear negative correlation with σ_1_, the size parameter associated with the common DGC/LGC and DBC/LBC bead types. Because four of the six CG beads in each monosaccharide are GC or BC types, σ_1_ strongly influences excluded-volume packing and therefore density. In contrast, *H*_v_ is most strongly correlated with ϵ_1_, indicating that cohesive interactions involving these common bead types dominate the *H*_v_. The remaining parameters show weaker correlations with the target properties, suggesting that they are either less influential within the sampled region or partially compensated by other parameters.

Before BUQ inference, all parameter values within the prescribed bounds were treated as equally likely through uniform priors. Conditioning on the target data produces posterior distributions that reveal which parameters are strongly constrained by the target properties and which remain weakly constrained. Specifically, ϵ_1_ and σ_1_ exhibit Gaussian-like unimodal posteriors, indicating strong sensitivity of the target properties to these parameters. In contrast, the unique-bead parameters, ϵ_2_, ϵ_3_, ϵ_4_, and σ_2_, σ_3_, σ_4_, retain broad, nearly uniform posteriors, reflecting lower sensitivity of the target properties to these parameters within the sampled range. This distinction identifies which FF parameters are tightly constrained by density and *H*_v_, and which would require additional structural or thermodynamic observables for further refinement.

Assessment of the surrogate model done using 100 random parameter sets sampled from the high-posterior-density region of the converged posterior distribution demonstrated strong agreement, as shown in Figure S6. This further confirmed that the surrogate models provide sufficiently accurate estimates for posterior exploration.

#### 3.5.2 Posterior Coverage Probabilities

Coverage probability analysis quantifies how well the posterior distributions are contained within the likelihood-defined confidence intervals for the target properties, providing a direct measure of whether Bayesian updating tightens, preserves, or loosens uncertainty relative to the likelihood. For a given confidence interval width, the coverage probability is defined as the fraction of the posterior probability mass contained within that interval.

Following Sose et al., the resulting curves are classified into three regimes. True-Unchanged posteriors closely follow the likelihood, producing coverage probability curves near the unity line.^36^ True-Tightened posteriors are narrower than the likelihood while remaining consistent with it, producing curves above the unity line. In this work, a posterior was classified as True-Tightened when uncertainty was reduced by more than 20% relative to the likelihood. True-Loosened posteriors are broader than the likelihood, producing curves below the unity line and indicating increased residual uncertainty. For FF development, True-Tightened behavior is desirable because the target data constrain the parameter space and improve predictive confidence while retaining parameter combinations that remain physically plausible.

Figure 11 shows the coverage probability curves for the posteriors of Figure 10, with each posterior approximated as a Gaussian centered at its mean. For *H*_v_, the curves for all three model monosaccharides rise rapidly to 100% coverage and lie above the unity line, classifying as True-Tightened. This indicates that Bayesian posterior characterization substantially reduces the range of parameter values capable of reproducing the target *H*_v_ data. The rapid increase in coverage probability also suggests that only a relatively narrow region of parameter space remains consistent with the target *H*_v_ values after Bayesian inference.

**Figure 11:**
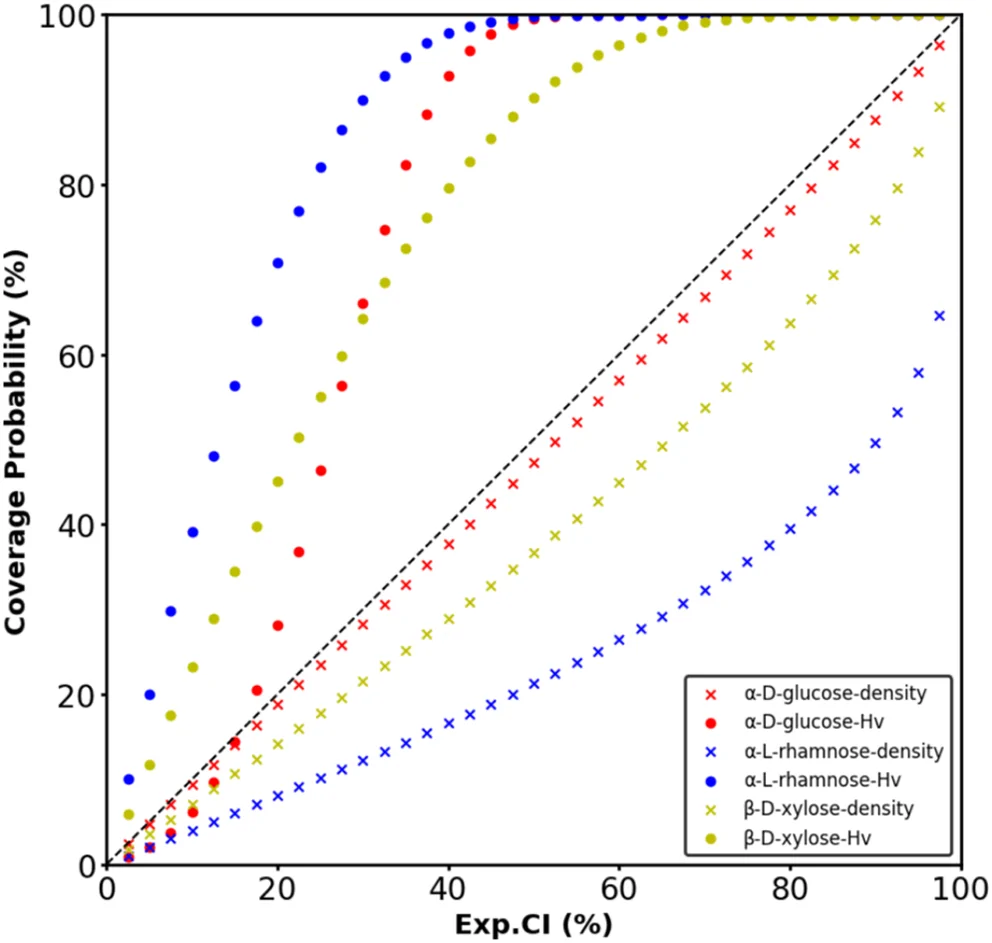
Coverage probability plotted against the target-property confidence interval for density and *H*_v_ of the three model monosaccharides. The dashed diagonal indicates 1:1 (likelihood-equivalent behavior), curves above, near, and below the line correspond to True-Tightened, True-Unchanged, and True-Loosened posteriors, respectively.

For density, the result is molecule-dependent. The α-d-glucose and β-d-xylose curves track the unity line, indicating True-Unchanged behavior. In these cases, BUQ preserves consistency with the target density data without substantially tightening or loosening the posterior. In contrast, the α-l-rhamnose density curve lies clearly below the unity line, indicating True-Loosened behavior and greater residual uncertainty in the density-relevant parameters for this molecule.

Overall, the coverage probability analysis demonstrates that BUQ provides a direct measure of FF reliability beyond agreement with target property values alone. This type of posterior coverage analysis is rarely reported in CG FF development and, to the best of our knowledge, has not been systematically applied to CG carbohydrate models, highlighting its value for assessing robustness and transferability in carbohydrate coarse-graining.

### 3.6 Robust Credible Intervals of Parameters

In CG FF development, optimized parameters are typically reported as a single best-fit set.^20,30–33^ However, many alternative parameter combinations can often reproduce the target properties within their associated uncertainties. Reporting only a single optimum therefore provides little information about the extent to which individual parameters can be varied while maintaining acceptable model performance. This limitation is particularly relevant when adapting an FF to new chemical environments, extending it to larger molecular systems, or improving compatibility with existing models. Although BUQ captures this information through posterior parameter distributions, these distributions do not directly indicate the allowable variation of individual parameters around the optimum. Consequently, practical parameter ranges that preserve agreement with the target data are rarely reported.

To address this gap, we performed an additional analysis after Bayesian inference to identify robust parameter ranges around the MAP estimate. We define a robust range as the interval over which independent perturbation of a single parameter does not move the predicted target property outside the 95% confidence interval of the target value, approximated as μ ± 2σ. This analysis complements the global sensitivity analysis in Section 3.4, Sobol indices identify which parameters most strongly influence each property, whereas robust ranges quantify how far individual parameters can be varied before predictive accuracy degrades.

The protocol was guided by the posterior-coverage results in Section 3.5.2. Only properties for which the model achieved approximately 100% coverage probability were included in the robust-range determination; therefore, the density of α-l-rhamnose was excluded. Starting from the MAP estimates, random parameter sets were generated by perturbing individual parameters, and the perturbation magnitude was iteratively reduced until all predicted properties remained within their respective 95% confidence intervals. Each sampling step used 10,000 × *S* samples, where *S* is the number of parameters. Parameters with broad, nearly uniform posterior distributions, ϵ_2_, σ_2_, ϵ_3_, σ_3_, ϵ_4_, and σ_4_, tolerated perturbations of 5%. In contrast, ϵ_1_ and σ_1_, which exhibit Gaussian-like posterior distributions and stronger property correlations, required smaller perturbations of 1.5% to satisfy the criterion. The resulting robust parameter ranges, associated property ranges, and BUQ-derived MAP values are summarized in Table 2. The MAP parameter values lie within the identified robust ranges, as shown in the Min-Max columns of Table 2, and the BUQ-predicted MAP property values fall within the corresponding property intervals. This includes the α-l-rhamnose density, even though it was excluded from the robust-range determination. Table S8 further shows that the original PSO-optimized parameters also lie within the robust ranges and remain close to the MAP values. These results have two practical implications. For applications that do not require further parameter adjustment, the PSO parameters reported in the SI are sufficient to reproduce the density and *H*_v_ of the three model monosaccharides. For applications requiring refinement, such as introducing new bead types or extending the model to disaccharides, branched glycans, or polysaccharides, the robust ranges define a per-parameter envelope within which additional sampling or objective-function-based optimization can be performed while maintaining the calibrated target properties within their 95% confidence intervals.

**Table 2:** Permissible Ranges of Perturbed Parameters Are Shown in Min-Max columns, along with the BUQ-obtained MAP Parameters and Properties^a^,^b^

parameters	MAP	min	max
ϵ1	–0.6478	–0.6576	–0.6381
σ1	3.6428	3.5881	3.6975
ϵ2	–0.4339	–0.4556	–0.4122
σ2	3.7326	3.5460	3.9193
ϵ3	–0.7483	–0.7857	–0.7108
σ3	4.3982	4.1784	4.6183
ϵ4	–1.2694	–1.3328	–1.2059
σ4	4.2400	4.0282	4.4520

monosaccharide	properties	MAP	min	max
α-d-glucose	Density (g/cm^3^)	1.46	1.38	1.54
β-d-xylose		1.44	1.37	1.50
α-l-rhamnose		1.32	1.25	1.40
α-d-glucose	H_v_ (kcal/mol)	29.67	28.33	30.91
β-d-xylose		26.18	25.33	27.01
α-l-rhamnose		26.46	25.52	27.37

a Min and Max of properties predicted using the permissible range are shown in the Properties section. Note, *E*_min_ = −ϵ values are reported here.

b *H*_v_: heat of vaporization.

These robust ranges should be interpreted as conservative bounds for independent, single-parameter variation rather than as the full feasible parameter region. The full BUQ posterior can include correlated multi-parameter changes that preserve agreement with target properties even when individual parameters move beyond the ranges reported here. Such coupled variations are not explicitly explored in the present robust-range analysis.

To the best of our knowledge, robust per-parameter ranges of this kind have not previously been reported for CG carbohydrate FFs. Existing CG carbohydrate models typically provide a single optimized parameter set validated against selected target properties. By combining BUQ posteriors with explicit robust-range determination, the present framework provides a more transparent characterization of parameter uncertainty and supplies practical guidance for extending the model to larger carbohydrate systems without invalidating the underlying calibration.

### 3.7 Transferability Assessment

Table 3 compares the thermodynamic properties predicted by CG MD simulations of the validation monosaccharides with atomistic reference values. Density errors range from 0.67% to 2.72%, with the largest deviation observed for β-d-glucose. *H*_v_ errors range from 0.17% to 7.67%, with the largest deviation observed for α-d-xylose. The low standard deviations of the CG values across independent simulations indicate stable and reproducible sampling, while the relatively large standard deviations of the AA reference values reflect the spread across the three AA FFs rather than within-replicate noise.

**Table 3:** Density and *H*_v_ of validation monosaccharides at 300 K. CG MD simulation results are compared with average values obtained from three AA FFs

monosaccharides	density (g/cm^3^)			heat of vaporization (kcal/mol)		
	CG	AA^a^	%error	CG	AA^a^	% error
α-d-galactose	1.467 ± 0.001	1.440 ± 0.043	1.88	28.02 ± 0.08	29.12 ± 5.02	3.77
α-d-mannose	1.433 ± 0.001	1.422 ± 0.051	0.77	28.07 ± 0.07	29.73 ± 7.44	5.58
α-d-xylose	1.390 ± 0.001	1.427 ± 0.045	2.59	24.53 ± 0.16	26.57 ± 2.77	7.67
α-l-fucose	1.318 ± 0.001	1.340 ± 0.029	1.64	26.64 ± 0.06	25.28 ± 4.43	5.38
β-d-galactose	1.469 ± 0.001	1.436 ± 0.046	2.30	29.36 ± 0.11	29.65 ± 5.76	0.97
β-d-glucose	1.474 ± 0.002	1.435 ± 0.047	2.72	29.87 ± 0.08	30.34 ± 4.74	1.55
β-d-mannose	1.461 ± 0.002	1.430 ± 0.051	2.17	29.19 ± 0.05	29.14 ± 5.47	0.17
β-l-fucose	1.325 ± 0.001	1.342 ± 0.029	1.27	27.09 ± 0.07	25.77 ± 2.67	5.12
β-l-rhamnose	1.328 ± 0.001	1.337 ± 0.029	0.67	27.09 ± 0.07	25.72 ± 1.64	5.33

a AA indicates the average from three FFs, where three independent simulations were performed with each FF.

Bond, angle, and dihedral distributions for the validation monosaccharides were extracted from the last 20 ns of the CG MD trajectories and compared with the corresponding mapped AA distributions in Figures S7 to S11, together with the RDFs. OC values ranged from 0.05 to 0.84 for bond lengths, 0.10 to 0.94 for bond angles, 0.00 to 0.88 for dihedrals in d-configured monosaccharides, and 0.34 to 0.86 for dihedrals in l-configured monosaccharides, with several distributions in each category showing substantial overlap, defined as OC ≥ 0.5.^66^ Together, the thermodynamic and structural agreement indicates that the optimized CG bead parameters are transferable across pyranose monosaccharides not included in the parameterization procedure.

### 3.8 Validation of the CG Model via *T*_g_

To assess the predictive capability of the developed CG FF across temperatures, we evaluated its ability to reproduce the glass transition temperature (*T*_g_) of monosaccharide systems. CG MD simulations were performed to predict *T*_g_ for the 12 monosaccharide systems, treating the α and β anomers as distinct structures. The resulting *T*_g_ values are compared with available literature data in Table 4. Although experimental *T*_g_ values generally do not distinguish between anomeric forms, the CG simulations indicate that the differences between α and β anomers of the same monosaccharide are relatively small, typically within approximately 10 K. Therefore, anomer-resolved comparison with anomer-blind experimental data is reasonable and is not expected to introduce significant bias.^92^ Figure S12 shows the variation of specific volume with temperature obtained from the CG MD simulations.

**Table 4:** Glass Transition Temperatures, *T*_g_, of 12 Monosaccharide Systems Obtained from CG MD Simulations and Compared with Literature Data

monosaccharide	CG (K)	experimental *T*_g_ (K)
α-d-galactose	317.41 ± 2.22	Galactose: 303.15,^93^ 305.15,^94,95^ 311.15,^96^ 383.15 ^97^
β-d-galactose	319.37 ± 2.58	
α-d-glucose	308.64 ± 3.19	Glucose: 294.15,^85^ 302.15,^98^ 303.15,^82,98^ 304.15,^97,99^ 306.15,^100^ 309.15,^96^ 310.15,^82^ 311.15,^94,95^ 312.15 ^97^
β-d-glucose	312.65 ± 3.62	
α-d-xylose	280.70 ± 4.51	Xylose: 282.15,^97,101^ 286.15,^94,95^ 287.15 ^96^
β-d-xylose	291.68 ± 3.72	
α-d-mannose	307.07 ± 1.41	Mannose: 303.15,^97,101^ 304.15,^96^ 309.15 ^94^
β-d-mannose	317.58 ± 1.77	
α-l-fucose	297.52 ± 1.55	Fucose: 304.15 ^96^
β-l-fucose	296.68 ± 6.15	
α-l-rhamnose	275.09 ± 2.21	Rhamnose: 273.15,^96^ 300.15 ^94^
	289.39 ± 5.00	
β-l-rhamnose	293.46 ± 6.06	

For α-d-galactose and β-d-galactose, the CG predictions of 317.41 ± 2.22 K and 319.37 ± 2.58 K lie ∼6 to 8 K above the upper bound of the cluster of literature values reported between 303 and 311 K, although both predictions remain well below the higher reported value of 383.15 K. For α-d-glucose, the predicted *T*_g_ of 308.64 ± 3.19 K falls within the broad experimental range of 294 to 312 K, and β-d-glucose at 312.65 ± 3.62 K agrees with the upper end of this range. The CG predictions for the xylose anomers bracket the reported experimental range of 282 to 287 K. α-d-xylose underestimates the lower bound by approximately 2 K, while β-d-xylose overestimates the upper bound by approximately 5 K. α-d-mannose at 307.07 ± 1.41 K lies within the experimental range of 303 to 309 K, while β-d-mannose at 317.58 ± 1.77 K is approximately 8 K above the upper bound. For fucose, both anomer predictions, 297.52 ± 1.55 K for α-l-fucose and 296.68 ± 6.15 K for β-l-fucose, lie approximately 7 K below the single reported experimental value of 304.15 K. α-l-rhamnose and β-l-rhamnose predictions fall within or near the reported experimental range of 273 to 300 K.

To evaluate whether the robustness identified extends beyond the properties used in parameterization, additional simulations were performed using 10 representative parameter sets sampled from the BUQ-derived robust ranges (Table S9). The resulting *T*_g_ values obtained for 12 monosaccharides remained comparable to those obtained using the PSO-derived parameter set and showed good agreement with the available experimental ranges (Figure 12). This indicates that the admissible parameter region identified from the training properties also provides stable predictions for *T*_g_.

**Figure 12:**
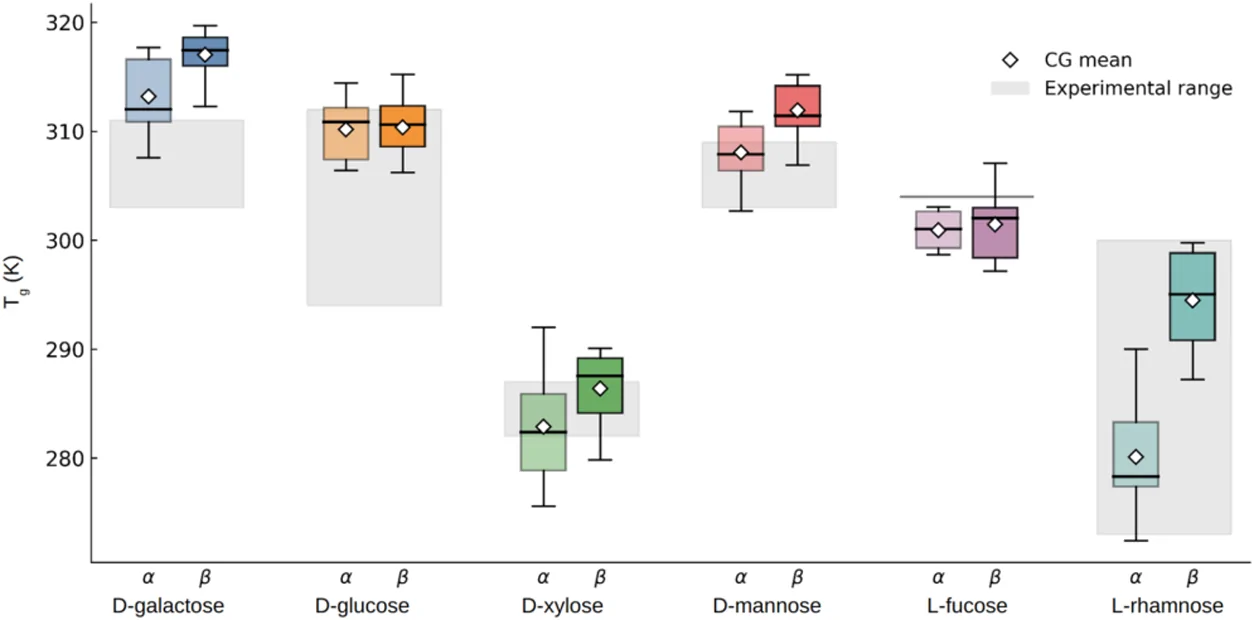
Box plots showing the distribution of *T*_g_ predicted using parameter sets sampled from the BUQ-derived robust ranges compared with experimental ranges.

Overall, the CG predictions fall within or near the reported experimental ranges for most monosaccharides. This level of agreement is notable because *T*_g_ was not used as a target property during parameter optimization. *T*_g_ depends on the same bulk cohesive energetics and molecular packing that determine *H*_v_ and density, therefore, its successful reproduction follows naturally from the bulk parameterization and provides an independent indication of the model’s transferability and predictive capability.

### 3.9 Surface Energy: Limitations of the Bulk-Optimized CG Model

Surface energy provides an independent test of whether the CG parameterization, optimized against bulk thermodynamic targets, density and *H*_v_, transfers to interfacial configurations.^102^ Surface energies of the model and validation monosaccharides were calculated from AA and CG MD simulations and are summarized in Table 5, results from the three individual AA force fields are reported in Table S10. Across the 12 monosaccharides, the CG model systematically overestimates AA-averaged surface energies by an average of approximately 65% (Table 5), demonstrating that the present bulk parameterization does not quantitatively reproduce surface energetics. The remainder of this section quantifies this limitation, identifies its physical origin through slab-bulk energy decomposition, and shows that the model nonetheless reproduces the stereochemistry-dependent ordering of surface energies observed in the AA references.

**Table 5:** Surface Energy (mJ/m^2^) of Bulk Monosaccharides from CG and AA MD Simulations

monosaccharide	CG	AA^a^	% error
α-d-galactose	142.95 ± 2.01	88.03 ± 8.53	62.38
α-d-glucose	138.06 ± 3.34	89.43 ± 12.64	54.37
α-d-mannose	142.02 ± 2.35	91.57 ± 16.30	55.09
α-d-xylose	133.83 ± 6.62	90.91 ± 10.49	47.21
β-d-galactose	133.88 ± 3.83	93.38 ± 17.10	43.38
β-d-glucose	137.70 ± 12.24	96.82 ± 6.52	42.23
β-d-mannose	133.83 ± 5.17	87.21 ± 16.06	53.46
β-d-xylose	134.12 ± 3.22	89.17 ± 5.04	50.42
α-l-fucose	127.96 ± 5.31	63.41 ± 11.76	101.79
α-l-rhamnose	127.75 ± 3.22	73.09 ± 7.04	74.78
β-l-fucose	123.58 ± 5.20	65.74 ± 2.93	87.98
β-l-rhamnose	131.57 ± 2.22	63.84 ± 6.84	106.10

a AA indicates the average from three FFs, where three independent simulations were performed with each FF.

CG-predicted surface energies range from approximately 124 to 143 mJ/m^2^, while the AA-averaged references span approximately 63 to 96 mJ/m^2^. The percentage errors range from approximately 42% for β-d-glucose to over 100% for α-l-fucose and β-l-rhamnose, with a mean error of approximately 65% across the 12 systems. Despite this quantitative overestimation, the CG model captures one chemically meaningful trend, the four l-configured deoxy monosaccharides, α-l-fucose, β-l-fucose, α-l-rhamnose, and β-l-rhamnose, consistently exhibit lower surface energies than the d-configured monosaccharides in both AA and CG simulations. In the AA results, the l-deoxy sugars cluster between approximately 63 and 73 mJ/m^2^, whereas the d-monosaccharides span approximately 87 to 96 mJ/m^2^. The CG model reproduces the same ordering. This trend is consistent with the molecular structures of fucose and rhamnose, which are deoxy sugars with one fewer hydroxyl group than the corresponding hexoses. The reduced hydroxyl content lowers intermolecular attraction at the surface, leading to slightly lower surface energies relative to the other monosaccharides.^103^

To understand the origin of the overestimation, the total potential energy difference between the slab (*U*_slab_) and bulk (*U*_bulk_) systems was decomposed into bonded and nonbonded contributions. The bonded terms include bond, angle, and dihedral energies, while the nonbonded terms include electrostatic interactions, present only in the AA simulations, and LJ interactions. In both representations, the bonded energy differences between slab and bulk systems are small, contributing only tens of kcal/mol (Tables S11–S13). This indicates that formation of the vacuum interface does not substantially perturb the local intramolecular geometry of the monosaccharides.

In the AA simulations, the nonbonded terms account for essentially all of *U*_slab_ – *U*_bulk_ (Tables S14 and S15). In the CG simulations, the LJ contribution is approximately 1000–1230 kcal/mol per monosaccharide (Table S14), roughly 2.2 times the corresponding AA LJ contribution. Because the CG model lacks an explicit electrostatic component, the CG LJ term absorbs part of the cohesive contribution that is distributed between LJ and electrostatic interactions in the AA representation. The total CG nonbonded contribution is therefore approximately 1.6 times the AA total nonbonded contribution, with CG/AA ratios across the 12 sugars ranging from 1.32 to 1.91. This ratio closely matches the ratio of the predicted CG and AA-averaged surface energies, indicating that the overestimated nonbonded slab-bulk energy difference is the dominant source of the surface-energy overestimation.

The physical mechanism underlying this discrepancy follows from how the effective CG LJ interactions transfer from bulk to interfacial environments. In the bulk, each molecule is surrounded by neighboring molecules, and the optimized CG interactions reproduce the target cohesive behavior by construction. At the interface, however, surface molecules lose a fraction of their favorable intermolecular contacts to the vacuum. Because the CG nonbonded interactions are effective interactions that implicitly absorb missing electrostatic contributions, the resulting interfacial energetic balance may differ from the AA representation.^104,105^ Thus, a compensation that works for isotropic bulk cohesion does not necessarily transfer to anisotropic interfacial environments.^106^ Additional factors may also contribute. In the AA representation, surface molecules can undergo orientational relaxation, including rearrangement of hydroxyl-rich and less polar molecular regions, to reduce the interfacial free-energy penalty. The reduced orientational specificity of the CG bead representation limits this relaxation. Similarly, hydrogen-bond directionality and network rearrangement, which are represented explicitly in AA simulations, are averaged into isotropic LJ interactions in the present CG model.^107^

Surface energy, therefore, exposes an inherent limitation of an LJ-only nonbonded representation tuned primarily to bulk thermodynamic targets. Improving quantitative prediction of surface energetics would require expanding the parameterization beyond bulk observables, for example, by including surface energy, temperature-dependent surface tension, adsorption energies of small probe molecules, or contact angles on hydrophobic and hydrophilic substrates as additional training targets. It may also require modifications to the nonbonded representation, such as introducing selected partial charges or using anisotropic or directional interactions. The fact that the present model reproduces the qualitative ordering of l-deoxy versus d-hexose surface energies, despite overestimating the magnitudes, suggests that the bead mapping retains chemically meaningful stereochemical information, while the main limitation lies in the LJ-only nonbonded representation.

## 4 Conclusion

In this work, we developed a transferable coarse-grained (CG) force field (FF) for pyranose monosaccharides covering the α and β anomers of six representative sugars: glucose, galactose, mannose, xylose, fucose, and rhamnose. The model was parameterized using three model monosaccharides, α-d-glucose, β-d-xylose, and α-l-rhamnose, and then tested without further parameter adjustment on nine additional validation monosaccharides. Each monosaccharide was represented using a six-bead mapping scheme that preserves key geometric and stereochemical features, including axial and equatorial carbon environments. Using a hybrid bottom-up/top-down strategy, bonded parameters were derived from mapped all-atom (AA) trajectories, while nonbonded parameters were optimized using particle swarm optimization to reproduce density and heat of vaporization, *H*_v_. For the model monosaccharides, density and *H*_v_ errors were at most 2.5% and 3.4%, respectively. For the validation monosaccharides, the corresponding errors remained at most 2.7% and 7.7%. The model also reproduced many dominant bonded and intermolecular structural features observed in mapped AA reference distributions, supporting transferability across pyranose monosaccharides.

Another key contribution of this study is the integration of Bayesian uncertainty quantification (BUQ) into CG carbohydrate FF development. By combining Sobol sequence sampling, Gaussian process regression surrogate models, and ensemble slice sampling, we inferred posterior distributions over the CG parameters rather than reporting only a single optimized parameter set. These posteriors identify parameter-property correlations, distinguish strongly constrained parameters from weakly identifiable ones, and provide MAP estimates. Posterior coverage analysis showed True-Tightened behavior for *H*_v_ across all three model monosaccharides, while density exhibited True-Unchanged or True-Loosened behavior depending on the monosaccharide. We further translated the posterior information into robust per-parameter ranges around the MAP estimates, defining how far individual parameters can be perturbed while keeping predicted properties within their 95% confidence intervals. To the best of our knowledge, this combination of posterior characterization, coverage-probability classification, and robust parameter-range determination has not previously been reported for CG carbohydrate force fields.

The model was also evaluated using properties not included in parameterization. CG-predicted glass transition temperatures, *T*_g_, agreed with reported experimental values within or near the available experimental ranges for most monosaccharides, supporting the transferability of the bulk parameterization. In contrast, surface energy was systematically overestimated by approximately 65% on average relative to AA-averaged references, revealing a limitation of the LJ-only nonbonded representation for interfacial properties. Slab-bulk energy decomposition indicated that this error arises primarily from overcohesive CG LJ interactions at the vacuum interface, although the model still captured the qualitative trend that l-configured deoxy monosaccharides have lower surface energies than d-hexoses. Overall, the developed CG model provides a transferable and uncertainty-aware framework for simulating bulk properties of pyranose monosaccharides, while clearly identifying interfacial energetics as an area requiring additional physics or training targets. Future work will extend this framework to disaccharides, oligosaccharides, polysaccharides, and carbohydrate-water systems.

## Supplementary Material



## Data Availability

AA FF files, developed CG FF topology and parameter files, as well as several analysis codes used in this paper, are available to access using the following GitHub link: https://github.com/Deshmukh-Group/CG-FF-Carbohydrates. Any additional data from the current study is available from the corresponding author upon reasonable request.

## Notes

Monosaccharide mapping schemes and coarse-grained bead definitions, methods for structural-distribution and enthalpy of vaporization analyses, density and heat of vaporization data for all-atom simulations, PSO parameter ranges and optimized nonbonded and bonded parameters, timestep stability analysis, Sobol sensitivity analysis, GPR validation against molecular dynamics simulations and posterior parameter credible intervals, transferability assessment, and slab-bulk bond, angle, dihedral, Lennard-Jones, and electrostatic energy analyses (PDF)
