# Supplementary material for: Development and Bayesian Uncertainty Quantification of Transferable Coarse-Grained Models for Carbohydrates
Source: J Chem Theory Comput. 2026 Oct 8;22(20):10834–54. doi: 10.1021/acs.jctc.6c01202 (PMC13718543; doi:10.1021/acs.jctc.6c01202)
Supplement: Supplementary file 1 [file ct6c01202_si_001.pdf]

**Supporting Information**

# Development and Bayesian Uncertainty Quantification of Transferable Coarse-Grained Models for Carbohydrates

Parisa Farzeen[1], Soumil Y. Joshi[1], Abhishek T. Sose[1], and Sanket A. Deshmukh[1]*

[1] *Department of Chemical Engineering, Virginia Tech, Blacksburg, VA 24061, United States*

*Corresponding author

E-mail address: sanketad@vt.edu

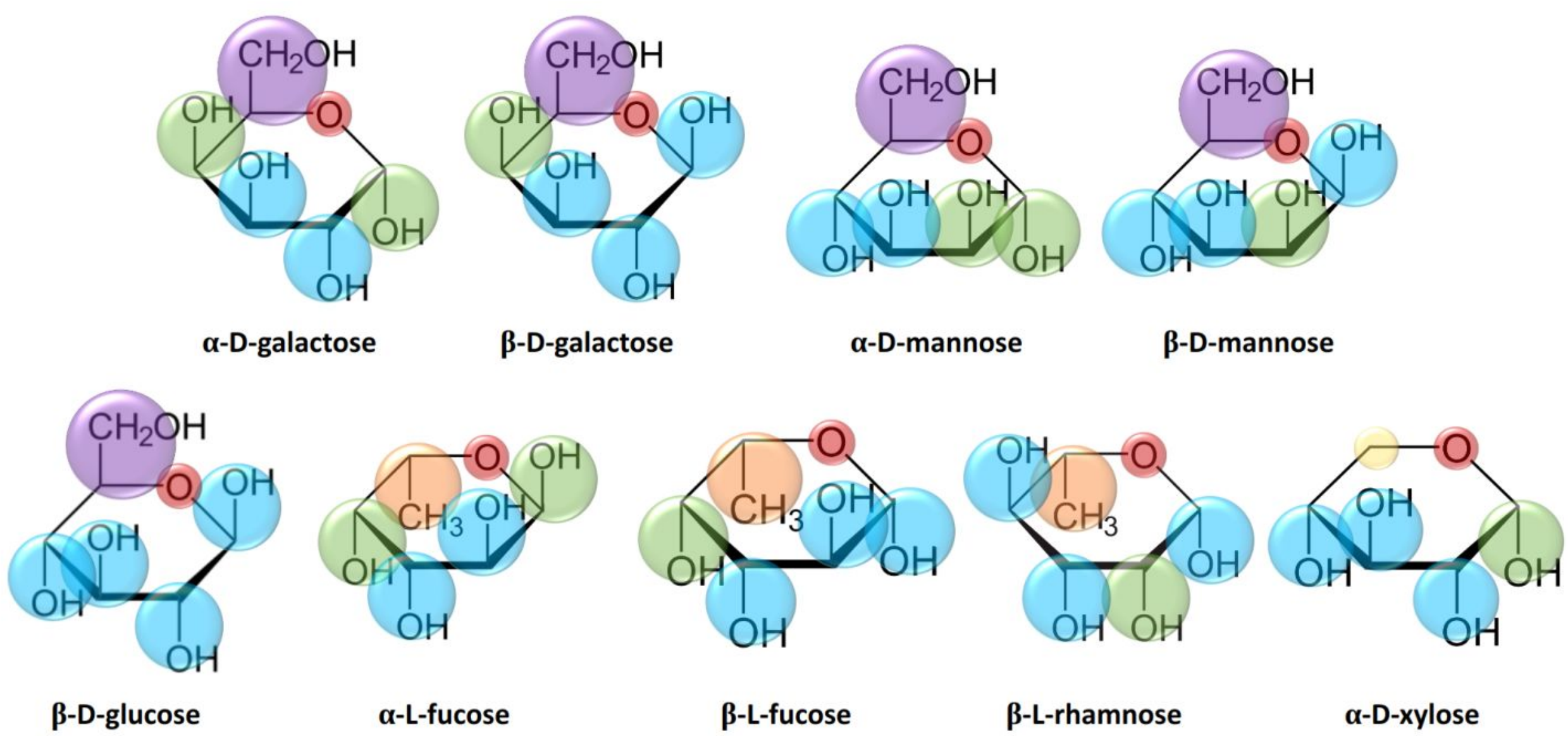


**Figure S1:** Mapping scheme for nine pyranose monosaccharides (validation monosaccharides).

**Table S1:** Six CG bead types used in monosaccharide models, the chemical groups they represent, and their masses. Prefixes D and L denote bead types associated with D- and L-stereoisomers, respectively.

| Bead name | Represented groups | Mass (g/mol) |
|---|---|---|
| DGC/LGC | >CH-OH | 30.0264 |
| DBC/LBC | >CH-OH | 30.0264 |
| DBB | >CH-CH2-OH | 44.0534 |
| LMB | >CH-CH3 | 28.0540 |
| DSB | >CH2 | 14.0270 |
| DRO/LRO | -O- | 15.9994 |

**Section S1: Methods of Analysis**

**S1.1 Structural Distributions**

Based on the mapping scheme from **Figure 2** in the main manuscript and **Figure S1**, the respective positions of the CG beads were obtained by determining the center of mass (COM) of the representative atoms, as shown in **Eq. S1**

$$r_{CG} = \frac{1}{M}\sum_{i=1}^{N} m_i r_i \quad \text{...............}\textbf{Eq. S1}$$

Where N and m are the number and total mass of atoms the CG be[1]ad constitutes, respectively, $r_i$ is the position of atom i, and M is the total mass of all the atoms grouped.[1] The mapped AA trajectory was used to determine the equilibrium bond lengths, angles, and dihedral angles, which were directly used for simulation without further optimization. Pair correlation functions between the COM of different CG beads were obtained to get an initial estimation of σ. The pair correlation distribution function was calculated using the following equation,

$$g(r) = \frac{\rho(\mathrm{r})}{\rho^{id}} = \frac{V}{N^2 4\pi r^2 d\mathrm{r}}\sum_{i=1}^{N} \Delta N_i(\mathrm{r}) \text{...............}\textbf{Eq. S2}$$

Where $\Delta N_i(r)$ is the number of beads at the distance of r, N is the total number of beads, and V is the total volume of the simulation box.

## S1.2 Enthalpy of Vaporization

The enthalpy of vaporization ($H_v$) is the change in enthalpy when one mole of liquid transforms into a gas under constant temperature conditions. This was calculated according to the method described in the work by Bejagam *et al*.[2], and using the following equation,[3]

$$\Delta H_v(\mathrm{T}) = \frac{1}{N_{mol}} E_{gas}(\mathrm{T}) - \frac{1}{N_{mol}} E_{liq}(\mathrm{T}) + \mathrm{RT} \quad \textbf{Eq. S3}$$

Where E is the total potential energy, R is the gas phase constant, T is the temperature, and $N_{mol}$ is the number of molecules, which is 1 for the NVT simulations.

**Table S2:** Density and Heat of vaporization of 12 monosaccharides from 3 different AA FFs.

| Monosaccharide | Density (g/cm$^3$) | | | Heat of vaporization (kcal/mol) | | |
|---|---|---|---|---|---|---|
| | CHARMM | GLYCAM | OPLS | CHARMM | GLYCAM | OPLS |
| α-D-galactose | 1.411±0.001 | 1.489±0.001 | 1.420±0.004 | 27.32±0.29 | 34.79±0.07 | 25.25±0.14 |
| α-D-glucose | 1.402±0.001 | 1.488±0.003 | 1.416±0.002 | 26.85±0.11 | 35.32±0.07 | 27.86±0.07 |
| α-D-mannose | 1.394±0.002 | 1.481±0.004 | 1.391±0.001 | 28.38±0.27 | 37.75±0.07 | 23.05±0.01 |
| α-D-xylose | 1.395±0.002 | 1.479±0.005 | 1.408±0.001 | 24.26±0.06 | 29.64±0.10 | 25.82±0.15 |
| α-L-fucose | 1.316±0.003 | 1.372±0.002 | 1.332±0.003 | 22.32±0.03 | 30.37±0.30 | 23.15±0.12 |
| α-L-rhamnose | 1.303±0.004 | 1.369±0.001 | 1.319±0.003 | 24.54±0.05 | 29.23±0.03 | 24.77±0.04 |
| β-D-galactose | 1.417±0.002 | 1.489±0.002 | 1.403±0.002 | 30.68±0.42 | 34.82±0.06 | 23.44±0.10 |
| β-D-glucose | 1.404±0.001 | 1.489±0.003 | 1.413±0.001 | 29.65±0.17 | 35.39±0.05 | 25.98±0.01 |
| β-D-mannose | 1.403±0.003 | 1.489±0.002 | 1.398±0.004 | 28.74±0.20 | 34.81±0.04 | 23.88±0.13 |
| β-D-xylose | 1.400±0.001 | 1.476±0.004 | 1.393±0.003 | 25.21±0.05 | 28.24±0.02 | 25.14±0.11 |
| β-L-fucose | 1.327±0.001 | 1.375±0.001 | 1.323±0.004 | 25.18±0.12 | 28.68±0.08 | 23.44±0.10 |
| β-L-rhamnose | 1.311±0.001 | 1.369±0.003 | 1.331±0.004 | 25.11±0.04 | 27.58±0.12 | 24.47±0.04 |

**Table S3:** Range of input nonbonded parameter values (min and max) used during PSO and the final optimized parameters obtained through PSO. Note, $E_{min} = -\epsilon$ values are reported here.

| CG bead | Parameter | Min | Max | Optimized |
| --- | --- | --- | --- | --- |
| DGC/LGC | -ϵ1 | -0.6250 | -0.6000 | -0.6181 |
| DBC/LBC | -ϵ1 | -0.6250 | -0.6000 | -0.6181 |
| DBB | -ϵ4 | -1.2600 | -1.1900 | -1.2100 |
| LMB | -ϵ3 | -0.8200 | -0.7400 | -0.7800 |
| DSB | -ϵ2 | -0.4500 | -0.3800 | -0.4200 |
| DGC/LGC | σ1 | 3.5100 | 3.7416 | 3.7060 |
| DBC/LBC | σ1 | 3.5100 | 3.7416 | 3.7060 |
| DBB | σ4 | 3.7416 | 4.0980 | 4.0624 |
| LMB | σ3 | 4.2762 | 4.4543 | 4.3653 |
| DSB | σ2 | 3.5278 | 3.6704 | 3.6508 |
| DRO/LRO | CHARMM ϵ (kcal/mol) -0.1000[4] | - | - | - |
| DRO/LRO | CHARMM $R_{min}/2$ (Å) 2.9399[4] | - | - | - |

Units: ϵ (kcal/mol) and σ (Å)

**Table S4:** Optimized bond parameters.

| Bond parameter | $k_b$ (kcal mol$^{-1}$ Å$^{-2}$ ) | $b_o$ (Å) |
| --- | --- | --- |
| DRO-DGC | 100 | 1.90 |
| DRO-DBC | 100 | 1.85 |
| DGC-DGC | 160 | 2.56 |
| DBC-DBC | 100 | 2.26 |
| DGC-DBC | 100 | 2.25 |
| DGC-DBB | 100 | 2.68 |
| DBC-DBB | 100 | 2.75 |

| | | |
|---|---|---|
| DBC-DSB | 100 | 1.98 |
| DRO-DBB | 100 | 2.24 |
| DRO-DSB | 100 | 1.49 |
| LRO-LGC | 100 | 1.90 |
| LRO-LBC | 100 | 1.85 |
| LGC-LGC | 140 | 2.56 |
| LBC-LBC | 100 | 2.26 |
| LGC-LBC | 100 | 2.26 |
| LGC-LMB | 100 | 2.31 |
| LBC-LMB | 100 | 2.31 |
| LRO-LMB | 100 | 1.88 |

**Table S5:** Optimized angle parameters.

| **Angle parameter** | **$k_\theta$ (kcal mol$^{-1}$ radian$^{-2}$ )** | **$\theta_o$(°)** |
|---|---|---|
| DRO-DGC-DGC | 65 | 70.39 |
| DRO-DBC-DGC | 100 | 78.61 |
| DRO-DGC-DBC | 100 | 97.48 |
| DRO-DBC-DBC | 100 | 98.93 |
| DGC-DGC-DBC | 46 | 90.07 |
| DBC-DGC-DBC | 100 | 117.24 |
| DGC-DBC-DBC | 100 | 100.37 |
| DBC-DBC-DBC | 100 | 117.26 |
| DGC-DBB-DRO | 100 | 63.73 |
| DBC-DBB-DRO | 100 | 77.76 |
| DBC-DSB-DRO | 100 | 127.49 |
| DGC-DRO-DBB | 100 | 130.80 |
| DBC-DRO-DBB | 100 | 161.23 |
| DGC-DRO-DSB | 100 | 103.71 |
| DBC-DRO-DSB | 100 | 135.99 |
| DBC-DBC-DBB | 100 | 123.85 |
| DBC-DGC-DBB | 100 | 122.73 |
| DBC-DBC-DSB | 100 | 100.60 |
| LRO-LGC-LGC | 26 | 70.42 |

| | | |
|---|---|---|
| LRO-LBC-LGC | 100 | 78.84 |
| LRO-LGC-LBC | 100 | 98.16 |
| LRO-LBC-LBC | 100 | 99.24 |
| LGC-LGC-LBC | 32 | 90.17 |
| LBC-LGC-LBC | 100 | 117.47 |
| LGC-LBC-LBC | 100 | 100.22 |
| LBC-LBC-LBC | 100 | 117.77 |
| LGC-LMB-LRO | 100 | 78.49 |
| LBC-LMB-LRO | 100 | 96.87 |
| LGC-LRO-LMB | 100 | 125.83 |
| LBC-LRO-LMB | 100 | 158.98 |
| LBC-LGC-LMB | 100 | 117.24 |
| LBC-LBC-LMB | 100 | 118.13 |

**Table S6:** Optimized dihedral parameters.

| Dihedral parameter | $k_\Phi$ (kcal/mol) | n | δ (°) |
|---|---|---|---|
| DBB-DRO-DBC-DBC | 3 | 1 | -261.24 |
| DBB-DRO-DBC-DGC | 3 | 1 | -292.88 |
| DBB-DRO-DGC-DBC | 1 | 2 | -292.05 |
| DBB-DRO-DGC-DGC | 2.6 | 1 | -301.54 |
| DBC-DBB-DRO-DBC | 3 | 1 | -93.96 |
| DBC-DBB-DRO-DGC | 1 | 2 | -112.04 |
| DGC-DBB-DRO-DBC | 3 | 1 | -81.97 |
| DGC-DBB-DRO-DGC | 1 | 2 | -86.73 |
| DBC-DBC-DBB-DRO | 3 | 1 | -181.64 |
| DBC-DGC-DBB-DRO | 3 | 1 | -244.74 |
| DBC-DBC-DBC-DGC | 3 | 1 | -206.78 |
| DBC-DGC-DBC-DBC | 3 | 1 | -248.25 |
| DGC-DBC-DBC-DGC | 3 | 1 | -235.85 |
| DGC-DGC-DBC-DBC | 5 | 1 | -251.06 |
| DBC-DBC-DBC-DBC | 3 | 1 | -174.75 |

| | | | |
|---|---|---|---|
| DBC-DBC-DGC-DBB | 3 | 1 | -107.83 |
| DBC-DBC-DBC-DBB | 3 | 1 | -185.82 |
| DGC-DBC-DBC-DBB | 3 | 1 | -155.78 |
| DRO-DBC-DBC-DBC | 3 | 1 | -174.79 |
| DRO-DBC-DGC-DBC | 3 | 1 | -111.36 |
| DRO-DGC-DBC-DBC | 3 | 1 | -118.63 |
| DRO-DGC-DGC-DBC | 3 | 1 | -84.17 |
| DBC-DBC-DSB-DRO | 3 | 1 | -214.42 |
| DBC-DBC-DBC-DSB | 3 | 1 | -174.03 |
| DBC-DSB-DRO-DBC | 3 | 1 | -109.25 |
| DBC-DSB-DRO-DGC | 3 | 1 | -97.17 |
| DSB-DRO-DBC-DBC | 3 | 1 | -225.49 |
| DSB-DRO-DGC-DBC | 3 | 1 | -271.87 |
| LBC-LBC-LGC-LGC | 3 | 1 | -109.21 |
| LGC-LBC-LBC-LGC | 2 | 1 | -124.08 |
| LBC-LGC-LBC-LBC | 2 | 1 | -112.09 |
| LBC-LBC-LBC-LGC | 2 | 1 | -156.36 |
| LGC-LBC-LBC-LMB | 2 | 1 | -204.92 |
| LBC-LBC-LGC-LMB | 2 | 1 | -245.21 |
| LBC-LMB-LRO-LBC | 2 | 1 | -229.16 |
| LBC-LMB-LRO-LGC | 2 | 1 | -241.55 |
| LGC-LMB-LRO-LBC | 2 | 1 | -260.48 |
| LGC-LMB-LRO-LGC | 2 | 1 | -270.73 |
| LBC-LBC-LMB-LRO | 2 | 1 | -176.32 |
| LBC-LGC-LMB-LRO | 2 | 1 | -115.92 |
| LMB-LRO-LBC-LBC | 2 | 1 | -127.29 |
| LMB-LRO-LBC-LGC | 2 | 1 | -100.75 |
| LMB-LRO-LGC-LBC | 2 | 1 | -77.82 |
| LMB-LRO-LGC-LGC | 5 | 1 | -71.40 |
| LRO-LBC-LBC-LBC | 2 | 1 | -183.47 |
| LRO-LBC-LGC-LBC | 2 | 1 | -248.42 |

| | | | |
|---|---|---|---|
| LRO-LGC-LBC-LBC | 2 | 1 | -240.36 |
| LRO-LGC-LGC-LBC | 4 | 1 | -275.82 |

**Section S2: MD simulations with different timesteps**

To demonstrate the numerical stability and timestep robustness of the CG model, we conducted CG MD simulations of the model monosaccharides using timesteps of 5, 8, and 10 fs on 100 randomly selected parameter sets. Results are shown in **Figure S2**.

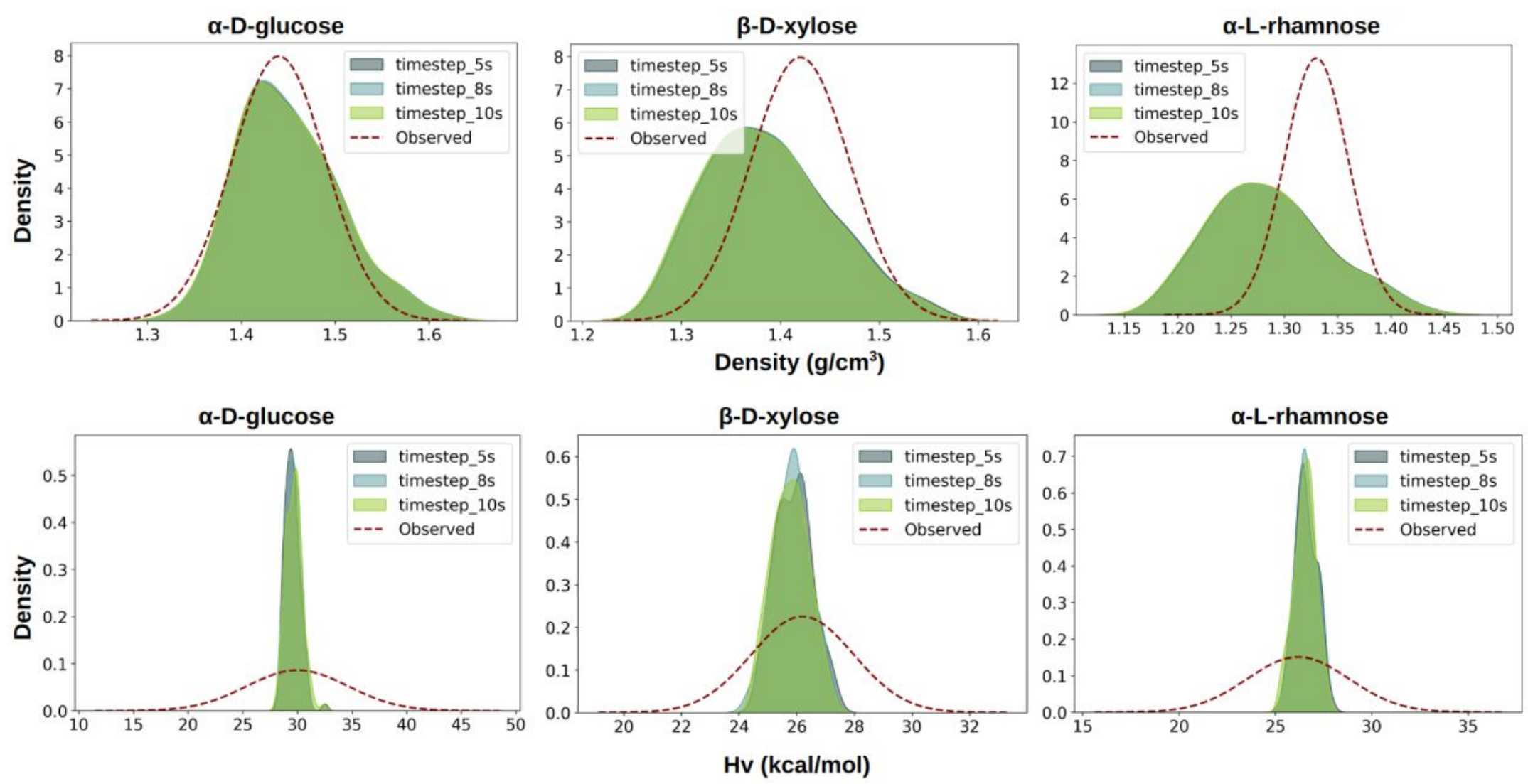


**Figure S2:** Comparison of distributions across different timesteps along with the observed distribution.

**Section S3: Gaussian Process Regression (GPR)**

In recent times, Gaussian Process Regression (GPR) has emerged as a promising surrogate model for MD simulations due to its flexibility and its capacity to offer probabilistic forecasts.[5] Since the GPR model relies on the covariance function (kernel) to define the relationship between data points, we compared multiple kernels using the k-fold cross-validation with 5 folds over 4000 data points. A total of five different kernels were compared: (1) Exponential, (2) Matern 32, (3) Matern 52, (4) Radial basis function (RBF), and (5) RBF+RBF. These kernels

were chosen as they define how the input parameters relate to the output properties in terms of smoothness, periodicity, or roughness.[6,7] The kernel with the highest average coefficient of determination was chosen as the covariance function for each property, and using that kernel, the surrogate model was trained on all 5000 data points.

### S3.1 Coefficient of Variation (CoV)

The coefficient of variation (CoV) is a statistical metric used to characterize the relative variability within a dataset. It is determined by dividing the standard deviation (σ) by the mean (μ) and expressed as a percentage. This provides a normalized measure of the dispersion of properties.

$$CoV(\%) = \frac{\sigma}{\mu} \times 100 \quad \text{............... Eq. S4}$$

**Table S7:** Mean, standard deviation, and coefficient of variation (CoV) for the three monosaccharides obtained from Sobol sensitivity data.

| Monosaccharide | Property | Mean (μ) | Standard deviation (σ) | CoV (%) | Best kernel ($R^2$) |
|---|---|---|---|---|---|
| α-D-glucose | Density | 1.46 | 0.06 | 4.11 | RBF (0.9998) |
| | $H_v$ | 28.07 | 0.76 | 2.71 | Mat32 (0.9414) |
| β-D-xylose | Density | 1.41 | 0.07 | 4.96 | Mat52 (0.9998) |
| | $H_v$ | 25.24 | 0.83 | 3.29 | RBF+RBF (0.9747) |
| α-L-rhamnose | Density | 1.29 | 0.06 | 4.62 | RBF+RBF (0.9997) |
| | $H_v$ | 26.04 | 0.76 | 2.92 | Mat32 (0.9669) |

Density in g/cm$^3$ and $H_v$ in kcal/mol

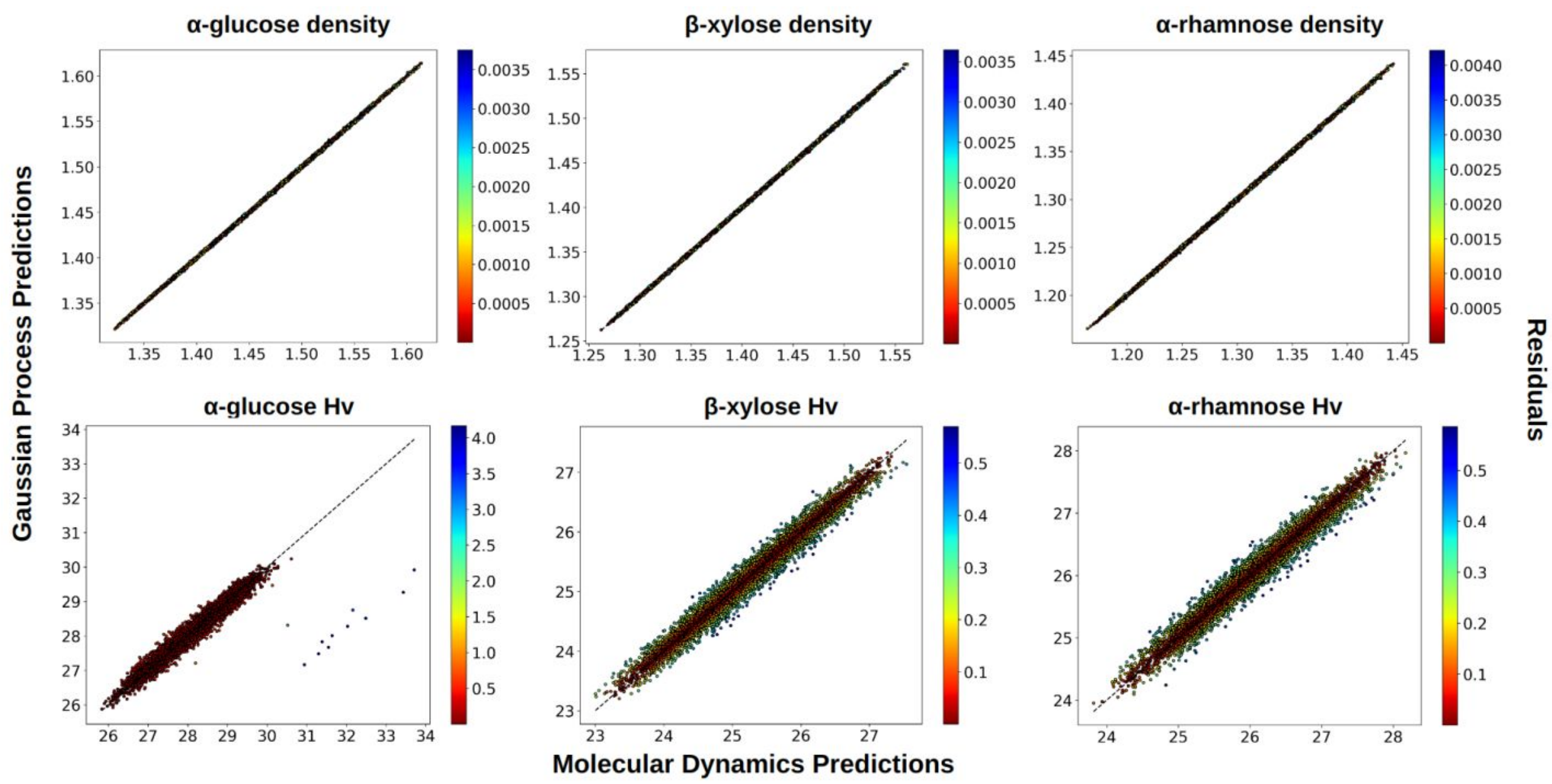


**Figure S3:** Parity plots for assessment of GPR model performance.

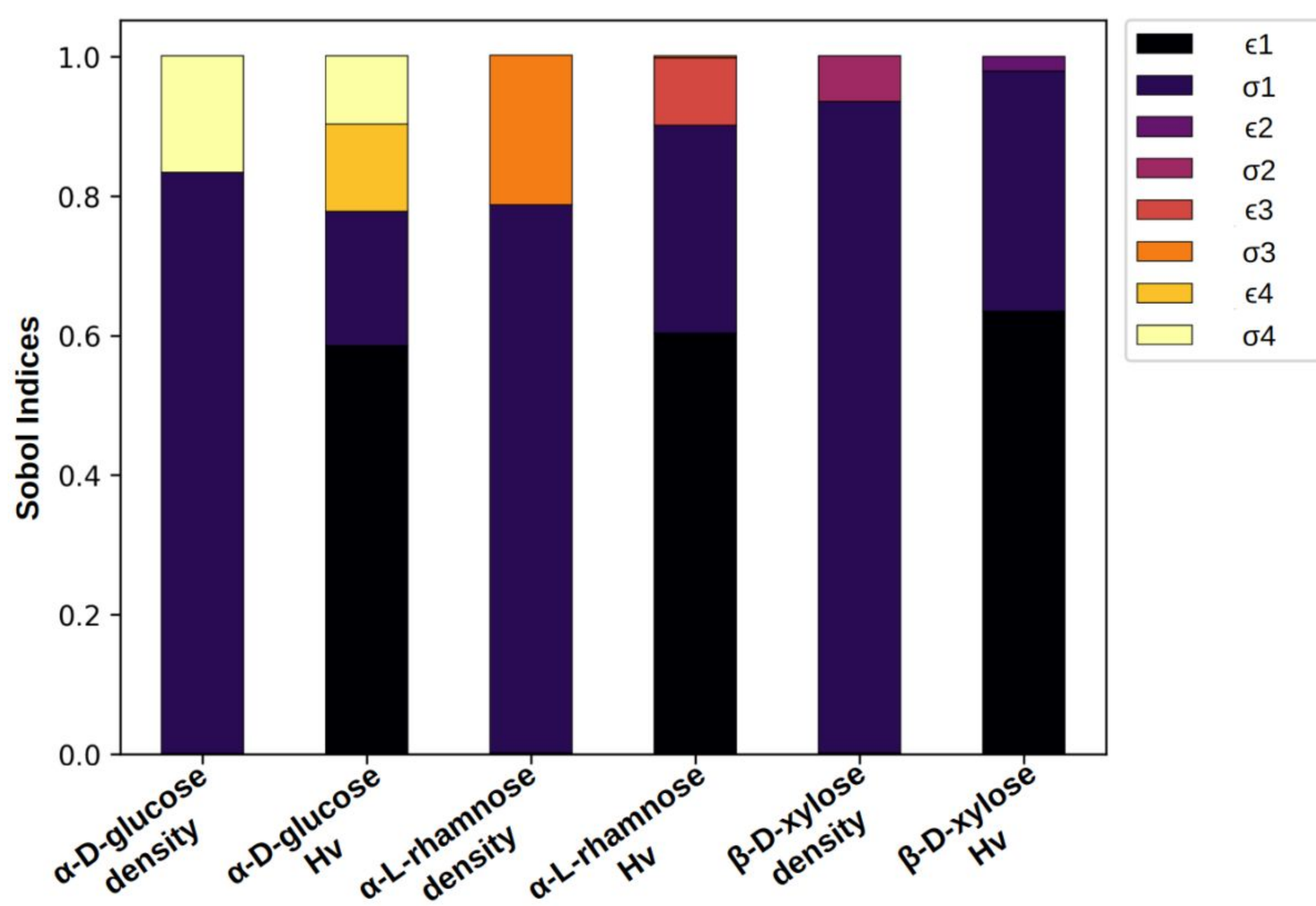


**Figure S4:** Sobol sensitivity indices indicating variation of properties being correlated with changes in FF parameters.

**Section S4: Convergence Metrics of ESS**

The integrated autocorrelation time (ACT) measures the statistical uncertainty due to sampling.[8] Since ensemble samplers generate correlated Markov chains, the number of independent samples must be estimated to assess how Monte Carlo error affects the results. In this work, we used the emcee package[9] to compute ACT. More details on the approach can be found in the article by Abhishek *et al.*[5] The ACT plot for combined parameters in **Figure S5** shows that ESS converged, and the parameters intersect with the 50 heuristic line.

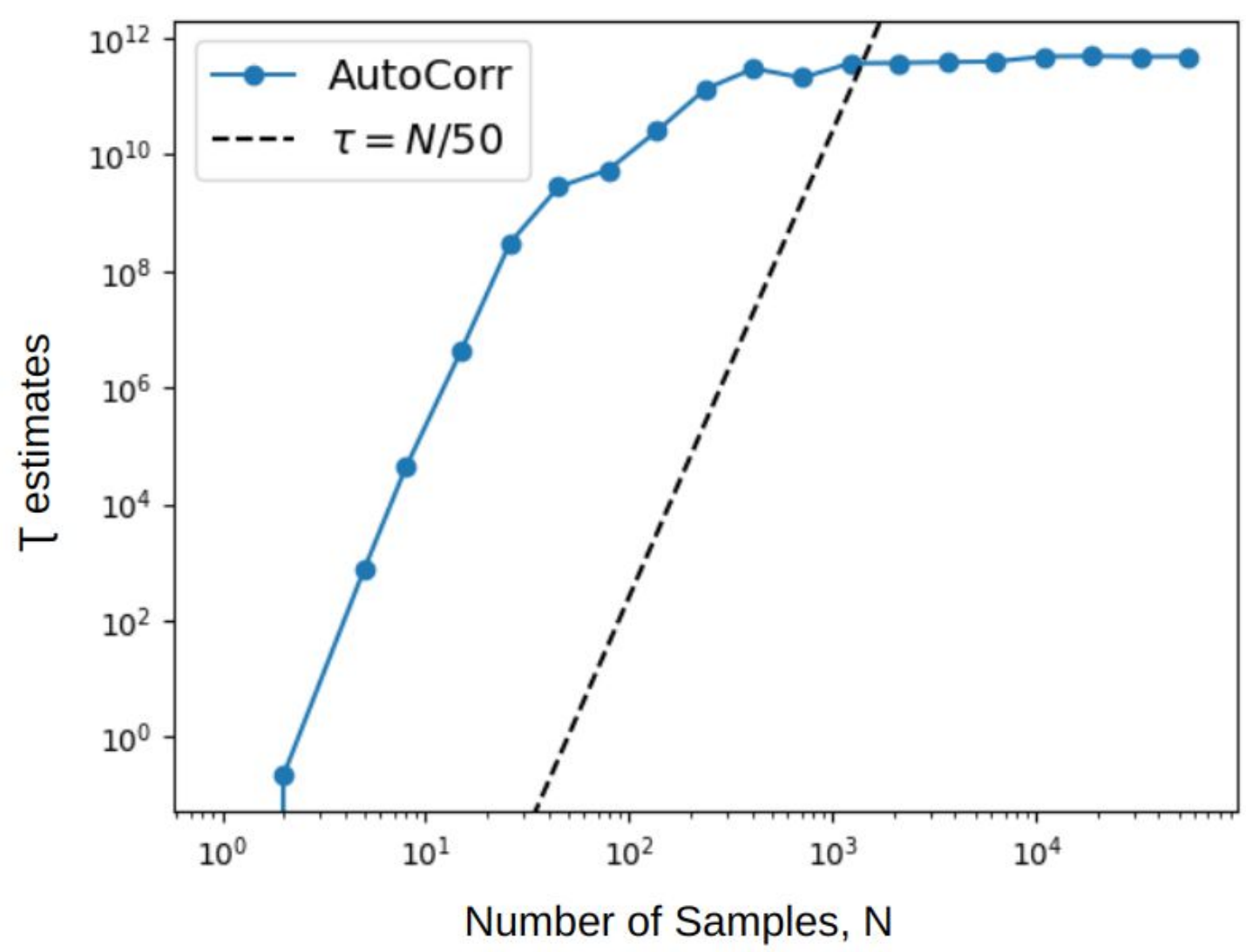


**Figure S5:** Autocorrelation time (ACT) graphs obtained for combined parameters, indicating when the Bayesian parameter estimation reached convergence.

**Section S5: Posterior Distribution Analysis**

**S5.1 Corner Plots**

Corner plots are a multi-panel visualization that shows the relationships between parameters and properties, as well as between parameters and parameters, and between properties and properties. It consists of scatter plots, histograms, and a correlation heatmap illustrating the behavior of Bayesian Uncertainty Quantification (BUQ) sampling. 100,000 samples

were randomly selected from the posterior distributions to create simplified representations of the samples.

Scatter plots were made using the densely sampled regions, meaning that the sampling algorithm frequently selected these parameter values. This suggests that these parameter combinations are more probable under the posterior distribution. Elongated and diagonal elliptical shapes suggest a high linear correlation, either positive or negative, depending on the orientation, between the two variables. The circular shapes indicate low linear correlation. Additionally, band-like patterns, with horizontal and vertical stripes, were observed. Generally, these indicate a conditional relationship in which one parameter is more deterministic while the other changes depending on the parameter space. More details of these shapes can be found in the article by Sose *et al.*[10] The histograms represent the posterior distribution of an individual parameter or property, where a wider distribution indicates higher uncertainty and a narrower distribution indicates higher confidence. The Pearson correlation coefficient highlights linear relationships within the parameter-property grid.

**Section S6: Gaussian Process Regression (GPR) model validation**

To further validate the GPR model predictions for the parameters from distributions, we performed MD simulations for the three model monosaccharides using randomly selected 100 FF parameter sets from the high posterior density region. Simulations were performed using similar methods detailed in **Section 2.1.2.2**. **Figure S6** shows the GP predictions and MD results for these 100 sets of parameters. This validation step ensured that the GPR model provided sufficiently accurate and reliable estimates of the target properties, validating its use in the broader sampling effort. As observed, there is good agreement between GP predictions and MD.

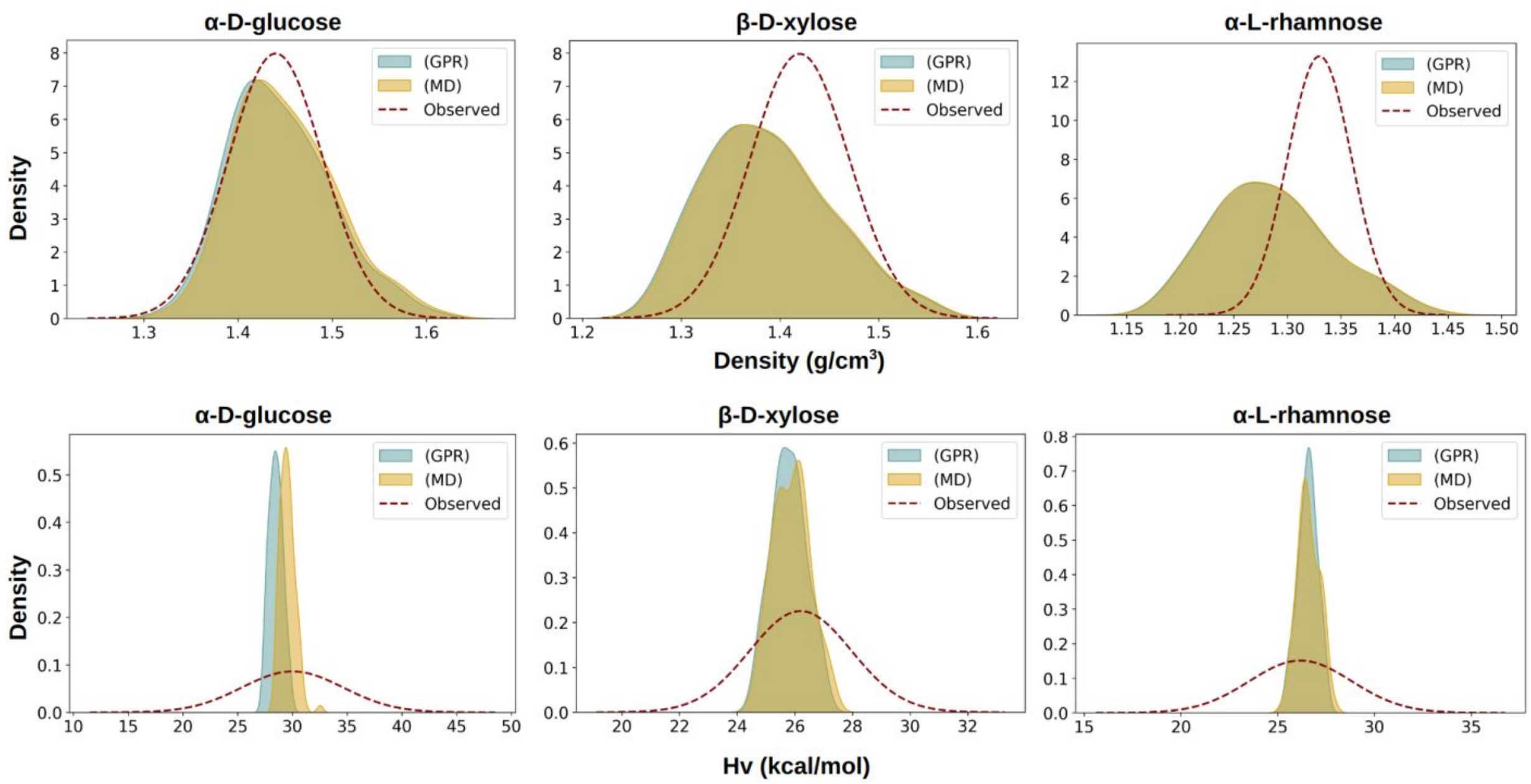


**Figure S6:** The comparison of GP predictions with the MD validation of 100 random posterior points for properties density and Heat of vaporization ($H_v$).

**Table S8:** A comparison of values defining the 95% credible intervals (CI) for parameters. The posterior distribution MAP represents the highest posterior probability, and the mean (μ_AP) represents the average parameter values obtained after Bayesian parameter estimation. The lower and upper limits indicate the 95% CI for the posterior distribution. PSO refers to the optimized parameters obtained using the PSO algorithm. The "Negative %" and "Positive % indicate the percentage difference between the lower and upper CI limits relative to the PSO value. Note, $E_{min}$ = -ϵ values are reported here.

| Parameter | MAP | μ_AP | | PSO | Lower CI | Upper CI | Negative % | Positive % |
|---|---|---|---|---|---|---|---|---|
| | | mean | stdev | | | | | |
| -ϵ1 | -0.6478 | -0.6266 | 0.0148 | -0.6181 | -0.6562 | -0.5971 | 6.16 | 3.40 |
| σ1 | 3.6428 | 3.7286 | 0.0962 | 3.7060 | 3.5364 | 3.6428 | 4.58 | 5.80 |
| -ϵ2 | -0.4339 | -0.4204 | 0.0121 | -0.4200 | -0.4446 | -0.3962 | 5.87 | 5.68 |
| σ2 | 3.7326 | 3.6499 | 0.1049 | 3.6508 | 3.4400 | 3.7326 | 5.77 | 5.72 |

| | | | | | | | | |
|---|---|---|---|---|---|---|---|---|
| -ϵ3 | -0.7483 | -0.7799 | 0.0224 | -0.7800 | -0.8248 | -0.7351 | 5.74 | 5.76 |
| σ3 | 4.3982 | 4.3596 | 0.1251 | 4.3653 | 4.1094 | 4.3982 | 5.86 | 5.60 |
| -ϵ4 | -1.2694 | -1.2138 | 0.0347 | -1.2100 | -1.2832 | -1.1445 | 6.05 | 5.42 |
| σ4 | 4.2400 | 4.0759 | 0.1156 | 4.0624 | 3.8445 | 4.2400 | 5.36 | 6.03 |

**Section S7: Transferability Assessment Results**

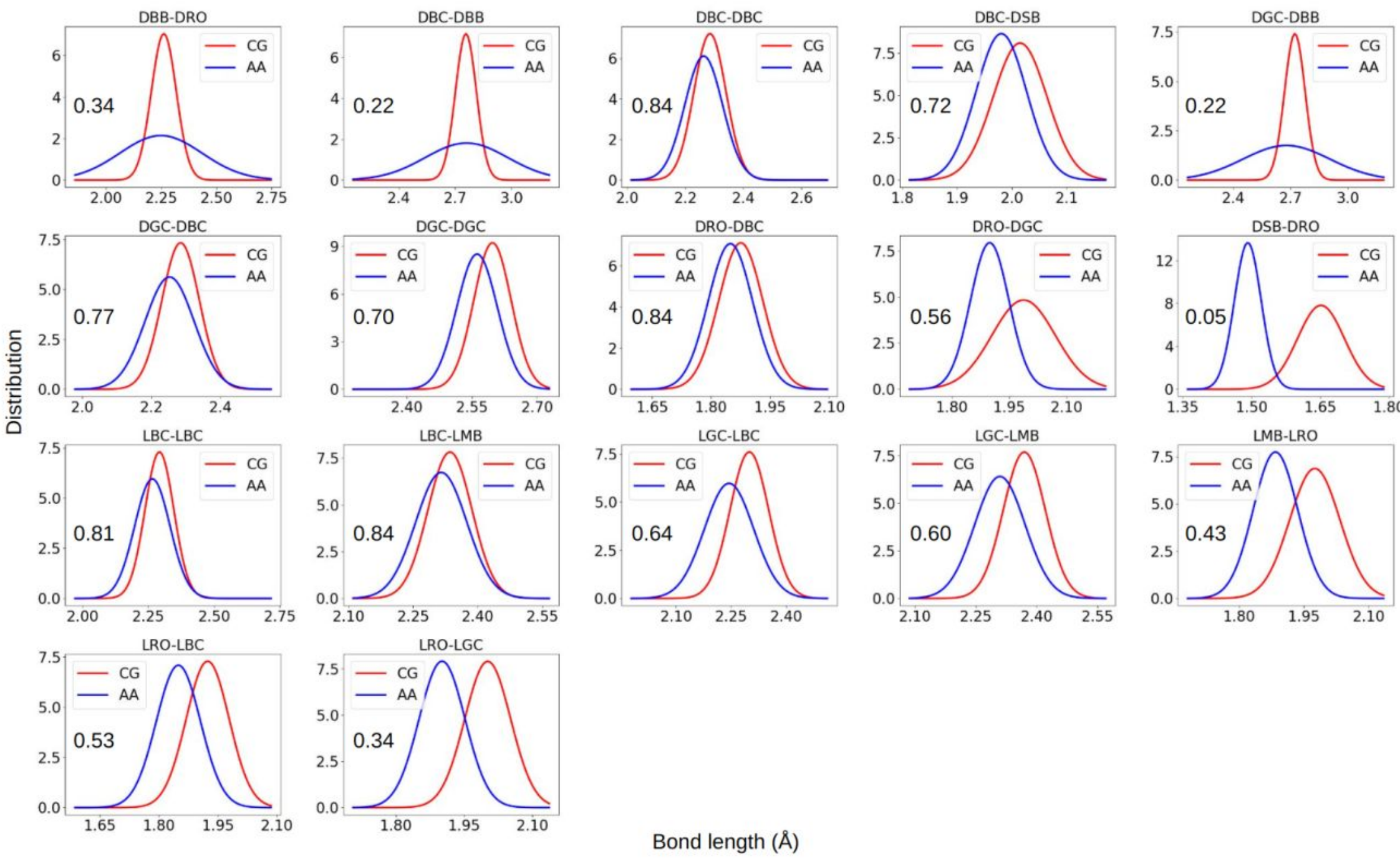


**Figure S7**: Distributions of bond lengths obtained from CG MD simulations of validation monosaccharides, and their corresponding mapped AA trajectories. CG bead names are listed in **Table S1** of the main manuscript.

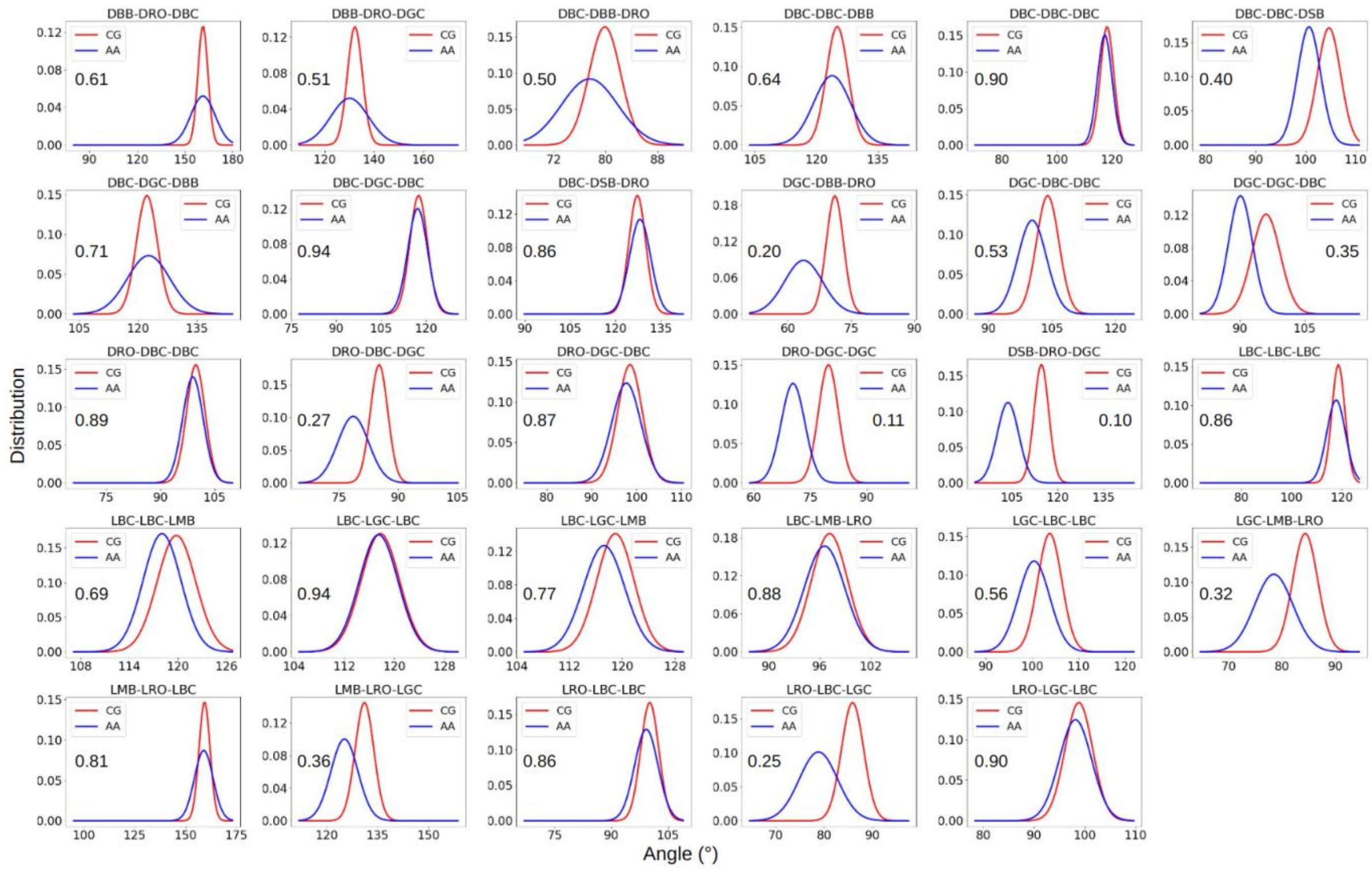


**Figure S8**: Distributions of angles obtained from CG MD simulations of validation monosaccharides, and their corresponding mapped AA trajectories. CG bead names are listed in **Table S1** of the main manuscript.

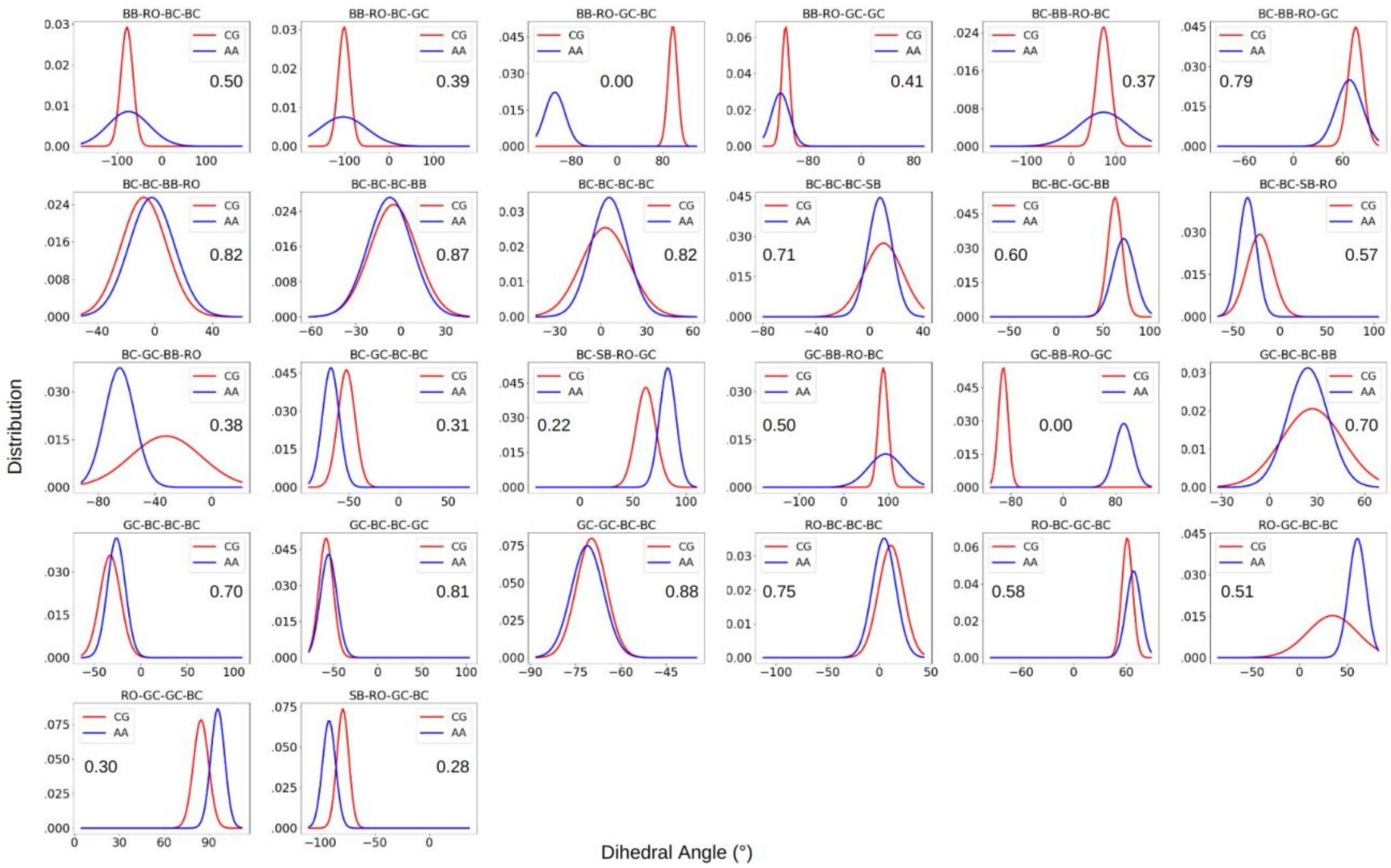

**Figure S9:** Distributions of dihedral angles for D-enantiomers obtained from CG MD simulations of validation monosaccharides, and their corresponding mapped AA trajectories. CG bead names are listed in **Table S1** of the main manuscript.

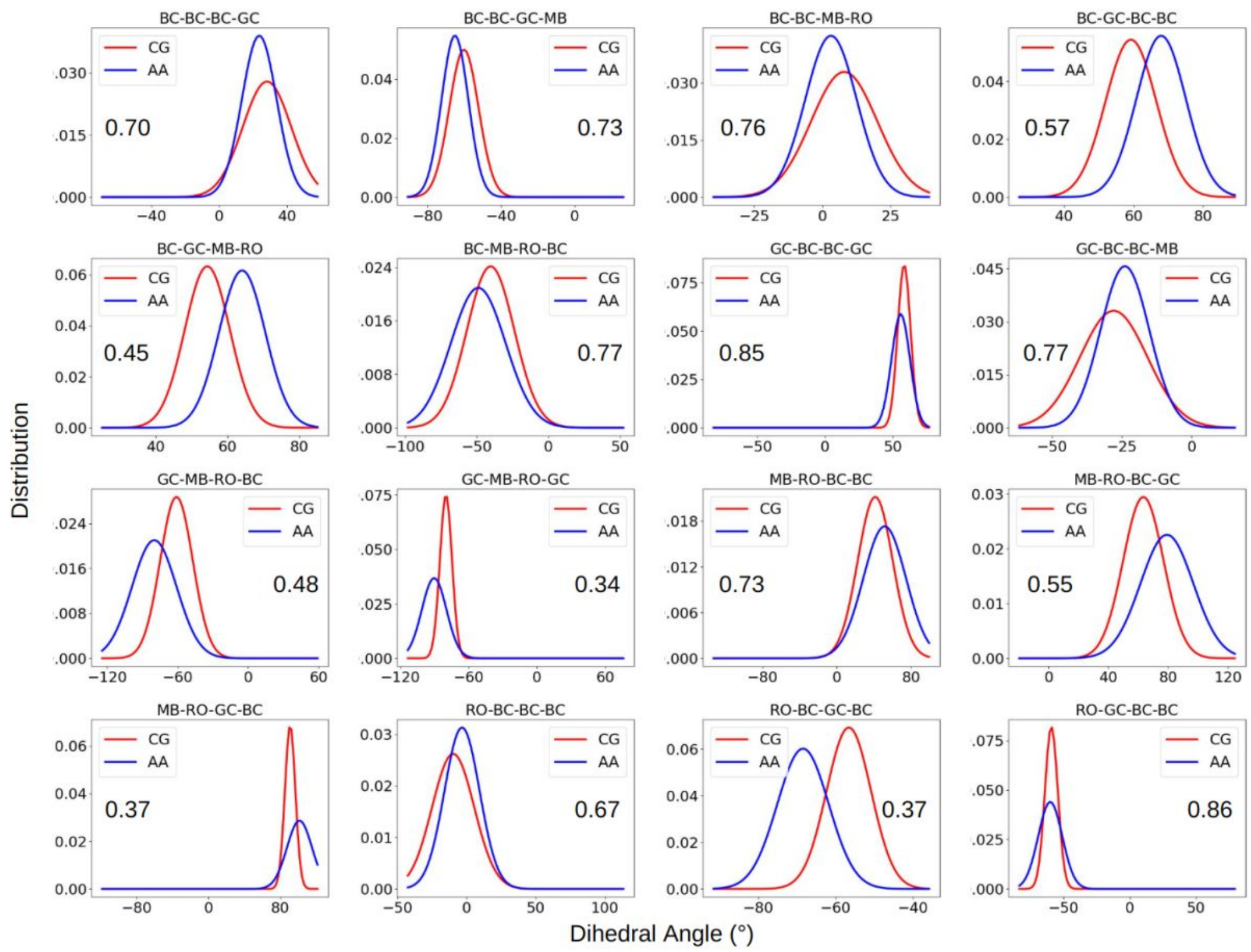


**Figure S10:** Distributions of dihedral angles for L-enantiomers obtained from CG MD simulations of validation monosaccharides, and their corresponding mapped AA trajectories. CG bead names are listed in **Table S1** of the main manuscript.

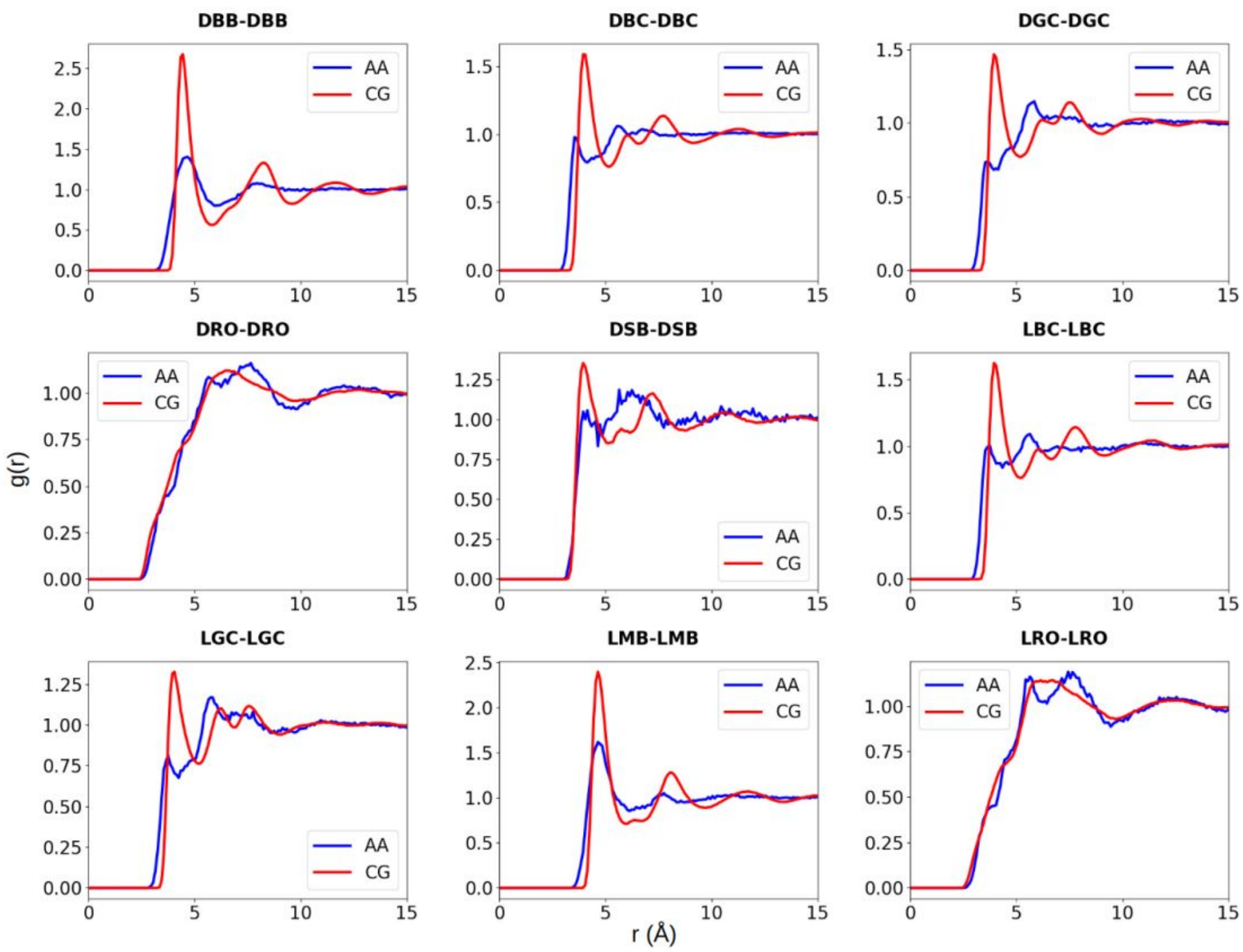


**Figure S11:** RDF obtained from CG (red) MD simulations of validation monosaccharides, and their corresponding mapped AA (blue) trajectories. CG bead names are listed in **Table S1** of the main manuscript.

## Section S8: Validation of the CG Model via $T_g$

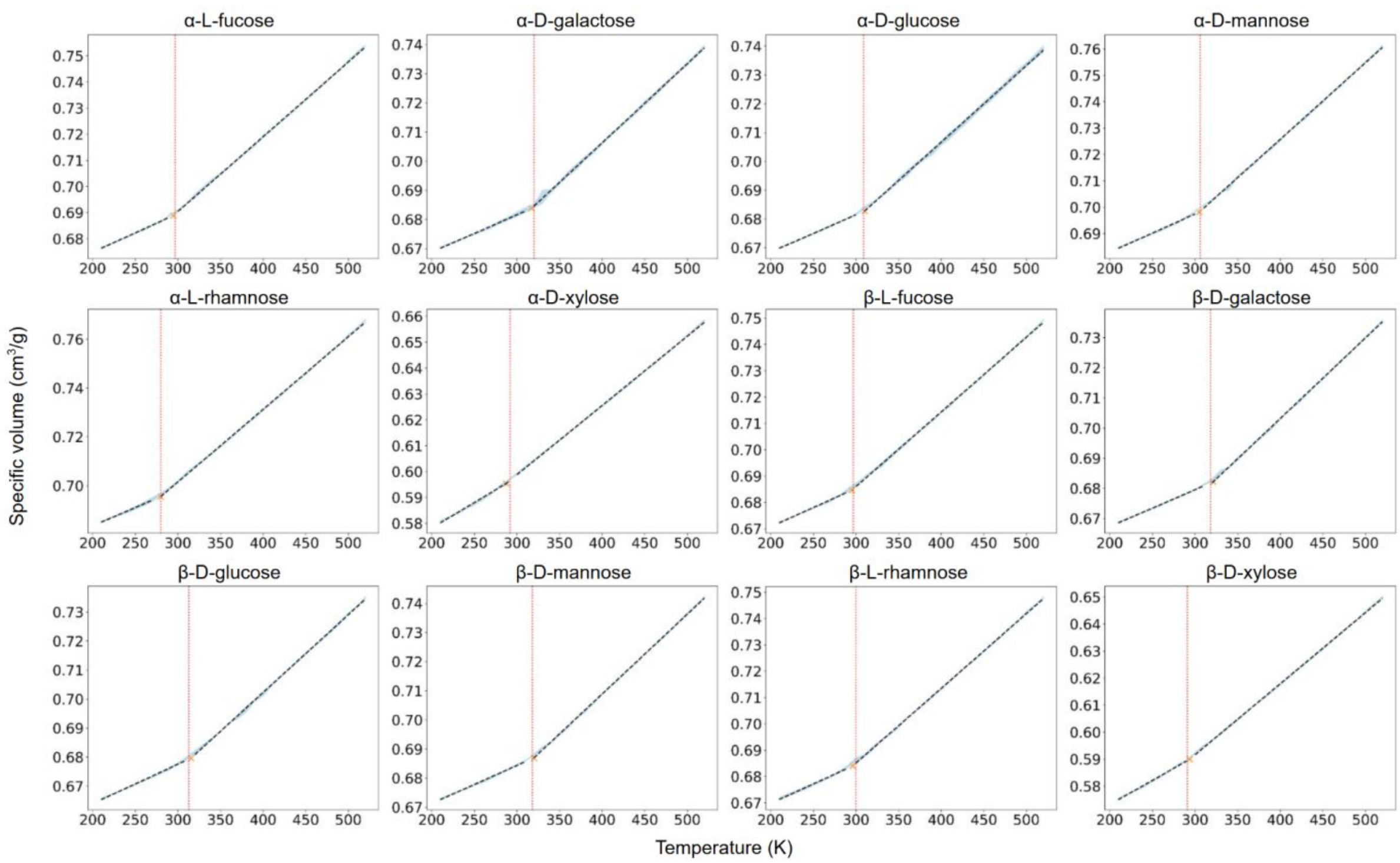


**Figure S12:** Specific volume as a function of temperature for model and validation monosaccharides. The black dotted line represents the mean specific volume averaged over three independent simulations, the red dotted line indicates the $T_g$, and the blue shaded region denotes one standard deviation.

**Table S9:** 10 representative parameter sets sampled from robust parameter ranges to obtain $T_g$. Note, $E_{min}$ = -ϵ values are reported here.

| Parameter sets | -ϵ1 | σ1 | -ϵ2 | σ2 | -ϵ3 | σ3 | -ϵ4 | σ4 |
|---|---|---|---|---|---|---|---|---|
| Set1 | -0.6440 | 3.6664 | -0.4179 | 3.7817 | -0.7606 | 4.3075 | -1.2349 | 4.1893 |
| Set2 | -0.6510 | 3.6388 | -0.4407 | 3.6739 | -0.7827 | 4.5794 | -1.2308 | 4.3911 |
| Set3 | -0.6451 | 3.5927 | -0.4239 | 3.6596 | -0.7248 | 4.3841 | -1.2757 | 4.2026 |
| Set4 | -0.6386 | 3.6472 | -0.4432 | 3.6797 | -0.7770 | 4.2433 | -1.3219 | 4.2509 |
| Set5 | -0.6453 | 3.6890 | -0.4348 | 3.6680 | -0.7380 | 4.3835 | -1.2089 | 4.1288 |
| Set6 | -0.6382 | 3.6966 | -0.4368 | 3.6028 | -0.7302 | 4.2313 | -1.3078 | 4.0401 |
| Set7 | -0.6539 | 3.6226 | -0.4421 | 3.7457 | -0.7618 | 4.4417 | -1.3066 | 4.2215 |

| | | | | | | | | |
|---|---|---|---|---|---|---|---|---|
| Set8 | -0.6485 | 3.6764 | -0.4547 | 3.8159 | -0.7469 | 4.3644 | -1.2691 | 4.2710 |
| Set9 | -0.6562 | 3.6897 | -0.4513 | 3.6224 | -0.7528 | 4.3196 | -1.3243 | 4.1750 |
| Set10 | -0.6388 | 3.6815 | -0.4310 | 3.6362 | -0.7267 | 4.5731 | -1.2930 | 4.2423 |

**Section S9: Validation of the CG Model via Surface Energy**

**Table S10:** Surface energy ($mJ/m^2$) of bulk monosaccharides from AA MD simulations with three FFs.

| Monosaccharide | CHARMM | GLYCAM | OPLS |
|---|---|---|---|
| α-D-galactose | 88.52±8.00 | 96.31±15.28 | 79.27±8.50 |
| α-D-glucose | 92.91±3.18 | 99.97±7.12 | 75.42±4.79 |
| α-D-mannose | 87.83±6.70 | 109.42±8.40 | 77.47±13.12 |
| α-D-xylose | 80.52±10.66 | 101.50±2.85 | 90.71±3.50 |
| β-D-galactose | 99.91±1.56 | 106.26±8.66 | 73.97±4.67 |
| β-D-glucose | 95.93±3.86 | 103.74±6.09 | 90.78±3.75 |
| β-D-mannose | 87.13±8.00 | 103.30±16.26 | 71.19±2.84 |
| β-D-xylose | 89.76±9.89 | 93.88±1.55 | 83.86±1.30 |
| α-L-fucose | 51.50±3.75 | 75.01±4.13 | 63.73±5.24 |
| α-L-rhamnose | 69.12±10.16 | 81.22±5.40 | 68.93±15.14 |
| β-L-fucose | 62.84±9.60 | 65.69±8.09 | 68.70±9.74 |
| β-L-rhamnose | 64.11±1.35 | 70.54±8.07 | 56.86±7.37 |

**Table S11:** Absolute bond energy differences (kcal/mol) between slab and bulk monosaccharides from AA and CG MD simulations.

| Monosaccharide | CHARMM | GLYCAM | OPLS | CG |
|---|---|---|---|---|
| α-D-galactose | 10.37 | 6.45 | 3.77 | 17.85 |
| α-D-glucose | 12.67 | 2.01 | 13.05 | 1.14 |
| α-D-mannose | 4.62 | 19.18 | 5.05 | 14.06 |
| α-D-xylose | 1.60 | 9.04 | 11.04 | 26.02 |

| | | | | |
|---|---|---|---|---|
| β-D-galactose | 2.75 | 3.18 | 5.72 | 2.16 |
| β-D-glucose | 9.65 | 6.32 | 5.61 | 1.52 |
| β-D-mannose | 2.81 | 1.07 | 0.57 | 8.34 |
| β-D-xylose | 0.08 | 5.52 | 7.29 | 17.54 |
| α-L-fucose | 3.05 | 9.64 | 6.25 | 29.43 |
| α-L-rhamnose | 3.05 | 8.57 | 3.05 | 24.81 |
| β-L-fucose | 3.19 | 8.83 | 11.28 | 20.05 |
| β-L-rhamnose | 4.93 | 1.31 | 7.90 | 17.81 |

**Table S12:** Absolute angle energy differences (kcal/mol) between slab and bulk monosaccharides from AA and CG MD simulations.

| Monosaccharide | CHARMM | GLYCAM | OPLS | CG |
|---|---|---|---|---|
| α-D-galactose | 18.22 | 16.57 | 13.43 | 13.21 |
| α-D-glucose | 26.41 | 10.94 | 16.94 | 15.84 |
| α-D-mannose | 16.67 | 6.19 | 36.17 | 5.10 |
| α-D-xylose | 30.18 | 31.75 | 4.74 | 9.14 |
| β-D-galactose | 53.36 | 25.79 | 5.06 | 14.52 |
| β-D-glucose | 49.49 | 0.44 | 14.98 | 6.69 |
| β-D-mannose | 31.81 | 25.50 | 16.52 | 13.66 |
| β-D-xylose | 17.07 | 10.45 | 3.55 | 7.81 |
| α-L-fucose | 19.28 | 11.62 | 32.81 | 13.64 |
| α-L-rhamnose | 26.53 | 15.53 | 13.93 | 9.84 |
| β-L-fucose | 25.67 | 18.18 | 1.13 | 13.24 |
| β-L-rhamnose | 28.03 | 21.21 | 4.08 | 8.13 |

**Table S13:** Absolute dihedral energy differences (kcal/mol) between slab and bulk monosaccharides from AA and CG MD simulations.

| Monosaccharide | CHARMM | GLYCAM | OPLS | CG |
|---|---|---|---|---|
| α-D-galactose | 16.34 | 17.91 | 16.86 | 3.08 |
| α-D-glucose | 5.76 | 25.50 | 52.17 | 18.85 |

| | | | | |
|---|---|---|---|---|
| α-D-mannose | 21.61 | 8.54 | 8.90 | 1.67 |
| α-D-xylose | 19.53 | 0.08 | 20.72 | 2.74 |
| β-D-galactose | 2.78 | 27.81 | 55.14 | 6.11 |
| β-D-glucose | 6.53 | 17.36 | 33.74 | 7.74 |
| β-D-mannose | 15.78 | 21.64 | 4.34 | 7.31 |
| β-D-xylose | 25.10 | 10.39 | 16.45 | 3.88 |
| α-L-fucose | 25.70 | 10.43 | 3.48 | 0.48 |
| α-L-rhamnose | 1.97 | 17.76 | 38.85 | 1.75 |
| β-L-fucose | 3.37 | 0.52 | 20.15 | 4.68 |
| β-L-rhamnose | 5.15 | 15.22 | 67.88 | 2.51 |

**Table S14:** Absolute LJ potential energy differences (kcal/mol) between slab and bulk monosaccharides from AA and CG MD simulations.

| Monosaccharide | CHARMM | GLYCAM | OPLS | CG |
|---|---|---|---|---|
| α-D-galactose | 505.04 | 632.08 | 440.70 | 1220.89 |
| α-D-glucose | 513.51 | 576.65 | 495.01 | 1221.16 |
| α-D-mannose | 506.79 | 577.54 | 506.73 | 1232.40 |
| α-D-xylose | 454.57 | 531.80 | 466.24 | 1042.56 |
| β-D-galactose | 475.43 | 626.46 | 503.42 | 1143.85 |
| β-D-glucose | 518.52 | 635.00 | 534.94 | 1194.52 |
| β-D-mannose | 544.77 | 611.11 | 484.13 | 1166.66 |
| β-D-xylose | 466.52 | 533.06 | 459.57 | 1044.75 |
| α-L-fucose | 478.68 | 507.69 | 469.80 | 1094.50 |
| α-L-rhamnose | 469.22 | 490.21 | 470.35 | 1106.51 |
| β-L-fucose | 499.27 | 535.10 | 429.67 | 1066.07 |
| β-L-rhamnose | 434.94 | 555.78 | 509.24 | 1129.11 |

**Table S15:** Absolute electrostatic energy differences (kcal/mol) between slab and bulk monosaccharides from AA MD simulations.

| Monosaccharide | CHARMM | GLYCAM | OPLS |
|---|---|---|---|
| α-D-galactose | 289.31 | 232.79 | 283.15 |
| α-D-glucose | 327.13 | 316.84 | 223.50 |
| α-D-mannose | 272.05 | 325.18 | 221.96 |
| α-D-xylose | 187.74 | 261.29 | 213.56 |
| β-D-galactose | 454.46 | 329.76 | 206.33 |
| β-D-glucose | 374.02 | 274.41 | 277.89 |
| β-D-mannose | 245.49 | 319.16 | 158.53 |
| β-D-xylose | 229.51 | 209.19 | 212.31 |
| α-L-fucose | 11.26 | 162.32 | 101.33 |
| α-L-rhamnose | 158.63 | 225.86 | 176.48 |
| β-L-fucose | 62.10 | 31.29 | 171.64 |
| β-L-rhamnose | 151.60 | 78.88 | 44.75 |

**References**

(1) An, Y.; Bejagam, K. K.; Deshmukh, S. A. Development of New Transferable Coarse-Grained Models of Hydrocarbons. *J Phys Chem B* **2018**, *122*, 7143–7153.
(2) Bejagam, K. K.; Singh, S.; An, Y.; Berry, C.; Deshmukh, S. A. PSO-Assisted Development of New Transferable Coarse-Grained Water Models. *J Phys Chem B* **2018**, *122*, 1958–1971.
(3) Wang, J.; Tingjun, H. Application of Molecular Dynamics Simulations in Molecular Property Prediction I: Density and Heat of Vaporization. *J Chem Theory Comput* **2011**, *7*, 2151–2165.
(4) Guvench, O.; Greene, S. N.; Kamath, G.; Brady, J. W.; Venable, R. M.; Pastor, R. W.; Mackerell, A. D., Jr. Additive Empirical Force Field for Hexopyranose Monosaccharides. *J Comput Chem* **2008**, *29*, 2543–2564.
(5) Sose, A. T.; Gustke, T.; Wang, F.; Anand, G.; Pasupuleti, S.; Savara, A.; Deshmukh, S. A. Evaluation of Sampling Algorithms Used for Bayesian Uncertainty Quantification of Molecular Dynamics Force Fields. *J Chem Theory Comput* **2024**, *20*, 5732–5742.
(6) 1.7. Gaussian Processes https://scikit-learn.org/stable/modules/gaussian_process.html (accessed Feb 4, 2025).
(7) Natsume. Gaussian Process Kernels - Towards Data Science - Medium https://medium.com/towards-data-science/gaussian-process-kernels-96bafb4dd63e (accessed Feb 4, 2025).
(8) Goodman, J.; Weare, J. Ensemble Samplers with Affine Invariance. *Comm. App. Math. Comp. Sci.* **2010**, *5*, 65–80.
(9) Foreman-Mackey, D.; Hogg, D. W.; Lang, D.; Goodman, J. Emcee: The MCMC Hammer. *arXiv [astro-ph.IM]* **2012**.
(10) Sose, A. T.; Gustke, T.; Bejagam, K. K.; Wang, F.; Savara, A.; Deshmukh, S. A. Development and Bayesian Uncertainty Quantification of Coarse-Grained Models of Metals

Based on Embedded Atom Method Potentials. *J Chem Theory Comput* **2025**, *21*, 12481–12496.
